# KIF11 promotes rheumatoid arthritis pathogenesis by activating M1 macrophage polarization and promoting inflammatory cytokine secretion

**DOI:** 10.1371/journal.pone.0347313

**Published:** 2026-05-13

**Authors:** Zhaonan Ban, Yongjie Ye, Hang Zhong, Zhengjiang Li, Zhangzhen Du, Wanquan Cao, Lei Yang, Shuxing Xing

**Affiliations:** Department of orthopedics, the Fifth People’s Hospital of Chengdu, Chengdu, Sichuan, China; Rutgers: Rutgers The State University of New Jersey, UNITED STATES OF AMERICA

## Abstract

**Background and Objective:**

KIF11, a mitotic kinesin, has been implicated in oncogenesis, but its specific role and mechanism in RA pathogenesis remain largely unexplored. We aim to explore functional role and molecular mechanism of KIF11 to promote RA progression.

**Methods:**

Bioinformatics analysis was first performed on public RA datasets (GSE55457, GSE55235, GSE2053, GSE12021, and GSE1919) to identify KIF11-associated DEGs and their enriched pathways using GO/KEGG and GSEA analysis. Lentivirus-mediated shRNA was employed to knock down KIF11 expression in MH7A cells and M1-polarized macrophages. The effects on macrophage activation were assessed by FACS for the surface marker CD86. The expression of inflammatory cytokines were measured by quantitative PCR and ELISA, respectively.

**Results:**

Bioinformatic analysis showd that KIF11-associated genes were significantly enriched in immune activation pathways, especially adaptive immune system and cytokine-cytokine receptor interaction. In vitro data demonstrated that KIF11 knockdown suppressed the M1 macrophage phenotype, with a marked decreased expression of CD86. Furthermore, KIF11 deficiency led to a profound decrease in the mRNA levels and protein secretion of cytokines in M1 macrophages.

**Conclusion:**

Depletion of KIF11 markedly inhibited the proliferation, migration, and inflammatory cytokine secretion in MH7A and attenuated the maturation of M1 macrophages, as well as the production of inflammatory cytokines, suggesting its pivotal role in RA pathology.

## Introduction

RA is a prototypical chronic systemic autoimmune disorder with persistent synovial inflammation, progressive joint destruction, and significant functional impairment. It predominantly affects small joints, but its systemic nature can result in manifestations beyond the musculoskeletal system, contributing to substantial morbidity [1]. Epidemiologically, RA affects approximately 0.5–1% of the adult population worldwide, with a higher prevalence in women and an increased incidence with advancing age. The disease imposes a substantial burden not only on individuals but also on healthcare systems due to its chronicity, progressive disability, and association with comorbidities such as cardiovascular disease and osteoporosis [2]. The heterogeneity in clinical presentation, disease course, and therapeutic response further underscores the complexity of RA and the pressing need for more precise diagnostic and prognostic biomarkers, as well as innovative therapeutic strategies [3].

Current research in RA pathogenesis has delineated pivotal roles for various immune cell subsets, such as B cells, macrophages, and fibroblast-like synoviocytes (FLSs), in perpetuating synovial inflammation and joint degradation [3]. Extensive investigations have elucidated the contributions of pro-inflammatory cytokines and their downstream signaling cascades in driving the inflammatory milieu of RA synovium [4,5]. Moreover, recent advances in genomic and transcriptomic profiling have found enormous differentially expressed genes implicated in RA, facilitating the discovery of potential biomarkers and therapeutic targets [4,6]. Despite these achievements, the focus has primarily centered on classical immune and inflammatory mediators, while the involvement of cell cycle-related proteins, particularly those regulating mitotic processes in synovial cells, remains underexplored [7,8].

Our previous research identified kinesin family member 11 (KIF11) as one of the ten hub genes in the pathogenesis of rheumatoid arthritis (RA) [9]. KIF11, a microtubule motor protein essential for mitotic spindle assembly and proper chromosomal segregation during cell division, has emerged as a molecule of interest [10]. KIF11 is upregulated in a variety of malignancies, where it facilitates tumor cell proliferation, migration, and resistance to therapy [11,12]. Notably, bioinformatics analyses of synovial tissue gene expression have identified KIF11 as a DEG in RA, suggesting a potential link between its mitotic functions and the pathological expansion of synovial fibroblasts [1]. Furthermore, machine learning-based analyses integrating multiple transcriptomic datasets have consistently highlighted KIF11 as a hub gene correlated with immune cell infiltration and inflammatory processes in RA synovium [3]. However, direct functional evidence delineating the role of KIF11 in the context of RA, particularly with regard to its impact on synovial cell behavior and inflammatory networks, remains lacking.

In the present study, we employed an integrative bioinformatics approach by analyzing multiple transcriptomic datasets to elucidate the molecular networks associated with KIF11 in RA. Importantly, these analyses are complemented by experimental validation using both clinical synovial tissue samples and established RA-derived fibroblast-like synoviocyte cell lines. The integration of multiple datasets and analytical modalities enhances the robustness and generalizability of the findings, while the incorporation of clinical samples ensures translational relevance. In summary, the overarching objective of this study is to clarify the functional involvement and molecular mechanisms of KIF11 in RA pathogenesis, thereby evaluating its viability as a novel therapeutic target. This research aspires to broaden the current understanding of disease mechanisms and foster the development of innovative, targeted intervention strategies for patients with refractory or progressive RA [13]. By systematically characterizing the expression patterns, regulatory networks, and cellular consequences of KIF11 dysregulation in RA, this research aspires to broaden the current understanding of disease mechanisms and foster the development of innovative, targeted intervention strategies for patients with refractory or progressive RA.

## Materials and methods

### RA-related datasets

The datasets GSE55457, GSE55235, GSE2053, GSE12021, and GSE1919 were sourced from GEO. A comparative analysis of DEGs among the five datasets was conducted utilizing the Xiantao platform (https://www.xiantao.love/). The thresholds established for significance were |log2 (FC)| > 1 and an adjusted p-value of < 0.05. Common DEGs identified across these five datasets were subsequently visualized using a Venn diagram.

### GO/KEGG analysis and GSEA analysis

For GO/KEGG pathway enrichment analysis, we selected the GSE55235 and GSE55457 datasets, which utilize the same Affymetrix HG-U133A platform (GPL96), to identify overlapping differentially expressed genes for in-depth functional annotation. The other datasets (GSE2053, GSE12021, and GSE1919) were used for preliminary cross-validation of differential genes and subsequent validation of hub gene expression patterns to ensure the reproducibility of our findings across different cohorts.

### PPI network

The PPI network encompassing genes related to KIF11 was acquired from the STRING database (https://string-db.org/). To ensure the reliability of interactions, the network was constructed with a medium confidence interaction score threshold set at 0.40. The resultant PPI network was then imported into Cytoscape software (version 3.9.1) for advanced topological analysis. The top ten nodes were ranked using the Maximum Clique Centrality (MCC) method. To ascertain the most pivotal nodes within the network, the ten hub genes were identified based on their connectivity degrees through Cytoscape’s built-in plugins. The expression levels of these hub genes in RA tissues were quantified and presented as mean values ± standard deviation (SD).

### Cell lines

The human RA-FLS cell line MH7A cells were obtained from JENNIO Biological Technology (Guangzhou, China) and cultured in DMEM supplemented with 10% FBS at 37 °C in a 5% CO_2_ atmosphere. To simulate RA conditions, MH7A cells were treated with TNF-α (10 ng/mL; Cat. no. 14832962; Thermo Fisher Scientific Inc.) or IL-1β (10 mg/L; Cat. no. SRP3083; Sigma-Aldrich Corp., St. Louis, MO, USA) for a duration of 24 hours.

### GO/KEGG and GSEA analysis

GO/KEGG and GSEA Analysis were performed on KIF11-associated DEGs using the Xiantao platform. The criteria for GO and KEGG analyses were set at absolute log2 Fold change > 1 and adjusted p-value < 0.01. GSEA was executed based on DEGs identifiers and log2 Fold values. Gene set permutations were conducted 1000 times for each analysis, with the screening criteria defined as a false discovery rate (FDR) < 0.25 and p.adjust < 0.05.

### KIF11 shRNA lentiviruses and KIF11 overexpressed plasmid

KIF11 human shRNA lentiviral particles (Locus ID 3832, Catalog Number: TL311921V) and their corresponding control lentiviral particles (Catalog Number: TR30021V) were obtained from Origene Inc. These lentiviral particles contain four unique 29-mer target-specific shRNAs and have lentiviral titers exceeding 10^7^ TU/ml. The transduction of both KIF11 shRNA and control shRNA lentiviral particles was conducted following the protocol provided by Origene and as described [14]. Moreover, Human, KIF11 cDNA ORF clone (Cat: HG17895-UT) and the control vector p CMV3-untagged (Cat.No. CV011) were purchased from Sino Biological (Beijing,China). The overexpressed procedure was used according to the protocol.

### MTT assay

An MTT assay was conducted to assess cell viability, following previously established protocols. Specifically, MH7A cells were seeded in 96-well plates at a density of 5 × 10^3 cells per well in 100 μL complete medium. MH7A cells were infected with either KIF11 shRNA or control shRNA lentiviruses for 24 h, 48 h, and 72 h, respectively. After adherence, cells were infected with either KIF11 shRNA or control shRNA lentiviruses. At 24 h, 48 h, and 72 h post-infection, 10 μL of MTT solution (5 mg/mL) was added to each well and incubated for 4 hours. Subsequently, the culture medium was carefully removed, and 150 μL of dimethyl sulfoxide (DMSO) was added to each well to dissolve the formazan crystals. The optical density (OD) of each well was measured at a wavelength of 490 nm using a microplate reader.

### EdU incorporation assay

Cell proliferation was assessed using the 5-ethynyl-2’-deoxyuridine (EdU) incorporation assay with the Click-iT EdU Imaging Kit (Thermo Fisher Scientific, Cat# C10354) according to the manufacturer’s instructions. Briefly, MH7A cells were seeded in 96-well plates at a density of 5 × 10³ cells per well and infected with KIF11 shRNA or control shRNA lentiviruses the next day. At 24 h or 48 h post-infection, cells were pulsed with 50 μM EdU for 2 h at 37°C. Cells were then fixed with 4% paraformaldehyde in PBS for 15 min at room temperature, permeabilized with 0.5% Triton X-100 in PBS for 20 min, and washed with PBS. EdU incorporation was detected by incubating cells with the Click-iT reaction cocktail (containing Alexa Fluor™ 594) for 30 min at room temperature, protected from light. Nuclei were counterstained with Hoechst 33342 (5 μg/mL) for 10 min. Images were captured using a fluorescence microscope (Olympus IX73) at 100 × magnification.

### Migration assay

The migration assay was executed utilizing Boyden chambers, which incorporated Transwell membrane filter inserts (Catalog Number: 3422, Corning Costar). In brief, 3 × 10^4^ cells per well were plated in serum-free DMEM within the upper chambers of a 24-well Transwell chamber (8 μm pore size) for migration assays, followed by incubation for 24 hours and 48 hours, respectively. The MH7A cells adhering to the lower surface of the filter were subsequently fixed in a 10% formalin solution for 30 minutes and then stained with 0.1% crystal violet for another 30 minutes. The number of migratory cells was enumerated under a light microscope, with counts performed across five fields within a single chamber for three samples.

### Western blot analysis

The primary antibodies utilized in this study included the rabbit recombinant monoclonal KIF11 antibody [EPR12280−76] (ab181981, Abcam), NF-kappaB p65 (D14E12) Rabbit Monoclonal Antibody (Cat.No.8242), Phospho-NF-kappaB p65 (Ser536) (93H1) Rabbit Monoclonal Antibody (Cat.No.3033), Anti-alpha tubulin antibody (Cat.No. ab4074), Anti-Lamin B1 antibody (Cat. No. ab16048) and the β-actin antibody [SP124] (ab115777, Abcam). Cell lysates were prepared using an immunoprecipitation assay buffer (Beyotime, Cat.No. P0013). A total protein separation was conducted via 10% SDS-PAGE, and the procedure was following previously described [15]. Band visualization was achieved using enhanced chemiluminescence reagents (Pierce; Thermo Fisher Scientific, Inc.).

### ELISA assay

The assays for IL-1β (Cat.No. EHC002b.96), IL-6 (Cat.No. EHC007.96), TNF-α (Cat.No. EHC103a.48) and IL-8 (Cat.No. EHC008(H).96) were conducted using reagents obtained from Neobioscience corporation.

### RNA extraction and real-time PCR

THP-1 cells were treated with 150 nM phorbol myristate acetate (PMA) for 24 h to differentiate into M0 macrophages. The M0 macrophages were subsequently cultured in fresh serum-free medium supplemented with 20 ng/mL IFN-γ and 10 pg/mL lipopolysaccharide (LPS) for another 24 hours to generate M1 macrophages. M1 macrophages were then transduced with KIF11 shRNA lentiviral particles as well as control shRNA lentiviral particles for a period of 48 hours. Total RNA was extracted using the RNApure kit (Bioteke, China) and reverse transcribed utilizing MLV-reverse transcriptase (Invitrogen, USA). Real-time PCR was conducted with an ABI Prism 7500, comprising 35 cycles at 95°C for 10 seconds and 55°C for 30 seconds. All PCR reactions were performed in duplicate. The primers were shown in Supplement File 1.

### Statistical analysis

All the results were shown as the mean ± standard deviation (S.D.). Independent samples were analyzed using an independent samples t-test. One-way analysis of variation followed by Tukey’s post hoc test was used for comparisons between >2 groups. All the experiments were repeat twice in duplicates. The statistical significance was set at p < 0.05.

## Results

### KIF11 is significantly upregulated in rheumatoid arthritis synovial tissues and under inflammatory conditions

**Fig 1:**
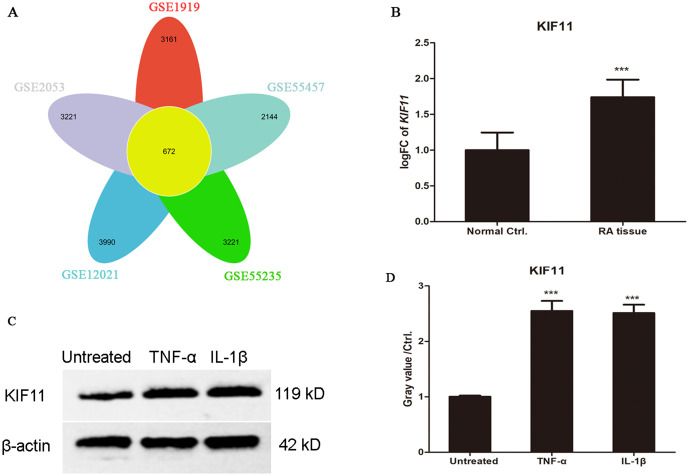
KIF11 is significantly upregulated in RA synovial tissues and inflammatory conditions. **(A)** A petal plot demonstrated 672 overlapping DEGs identified from five RA synovial tissue datasets (GSE55457, GSE55235, GSE2053, GSE12021, and GSE1919). DEGs were selected with thresholds of |log2FC| ≥ 0.5 and adjusted p-value < 0.05. **(B)** The mRNA expression levels of KIF11 in rheumatoid arthritis (RA) and normal control (NC) synovial tissues were analyzed in six independent GEO datasets: GSE1919, GSE12021, GSE55235, GSE55457, GSE77298, and GSE2053. Data are presented as mean ± SD. ***P < 0.001, compared with NC controls. **(C)** MH7A cells were treated with TNF-α (10 ng/mL) and IL-1β (10 ng/mL) for 24 h, and the expression of KIF11 was determined *via* western blotting. **(D)** The relative expression of KIF11 was shown in histogram. ***P < 0.01, compared with untreated group.

To systemically identify key drivers in the pathogenesis of rheumatoid arthritis (RA), we integrated and analyzed five independent GEO datasets of RA synovial tissues. The results showed that 672 consistently differentially expressed genes (DEGs) across all datasets, as illustrated in the Venn diagram (Fig 1A). Among these promising candidates, KIF11, a mitotic kinesin, was one of the hub genes in RA progression as we previously reported [9]. We first sought to validate the dysregulation of KIF11 at the transcriptional level. Comparative analysis confirmed that KIF11 mRNA level was significantly elevated in RA synovial tissues compared to normal controls across all five independent cohorts (Fig 1B), underscoring its consistent association with the disease.

Given that the RA synovial microenvironment is characterized by chronic inflammation, we hypothesized that pro-inflammatory cytokines might directly regulate KIF11 expression. Next, we treated the human synovial cell line MH7A with TNF-α and IL-1β. Strikingly, stimulation with these cytokines significantly induced the protein expression of KIF11 (Fig 1C and D). This finding suggests that the inflammatory milieu in RA joints may be a direct upstream trigger for KIF11 upregulation, pointing to a potential mechanistic link between inflammation and KIF11-driven pathology.

### Identification of KIF11-associated pathways in RA

**Fig 2:**
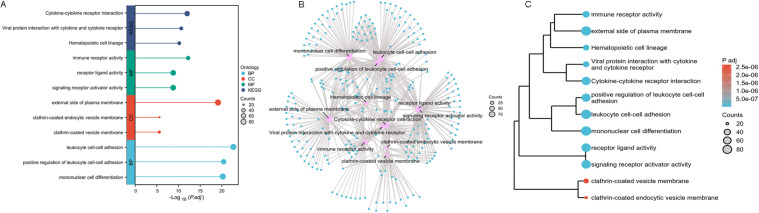
Functional enrichment analysis of 1350 overlapping differentially expressed genes (DEGs) in rheumatoid arthritis datasets of GSE55235 and GSE55457. **A.** Bubble plot of GO/KEGG enrichment analysis. The enrichment analysis results were visualized using the ggplot2 [version 3.4.4] package. **B.** Network visualization of enrichment analysis results. This was performed using the ggplot2, igraph, and ggraph packages [42]. **C.** GO/KEGG analysis cluster tree. The pairwise similarity of enriched terms was calculated using the Jaccard similarity index (JC). The results were then subjected to cluster analysis *via* hclust, and the clustering outcome was visualized using the ggplot2 [version 3.4.4] package.

To investigate the potential biological functions and pathways through which KIF11 might drive RA pathogenesis, we identified 1350 overlapping DEGs from the GSE55235 and GSE55457. These overlapping DEGs were subjected to a comprehensive functional enrichment analysis, including GO/KEGG pathway analyses. As visualized in the bubble chart (Fig 2A), the most significantly enriched GO terms were predominantly associated with immune and inflammatory processes. Key enriched biological processes (BP) included leukocyte cell-cell adhesion, mononuclear cell differentiation and the positive regulation of lymphocyte activation. In terms of cellular components (CC), the DEGs were significantly localized to the external side of plasma membrane, clathrin-coated endocytic vesicle membrane, and clathrin-coated vesicle membrane. MF analysis highlighted enrichments in immune receptor activity and signaling receptor activator activity. Concurrently, KEGG pathway analysis revealed a strong involvement of these genes in critical immune-related pathways.

To elucidate the interrelationships between these enriched terms, we constructed a network diagram (Fig 2B). This visualization clearly demonstrates that immune system processes form a central, highly interconnected cluster, with terms like “cytokine-cytokine receptor interaction”, “leukocyte cell-cell adhesion”, “receptor ligand activity” and “mononuclear cell differentiation” serving as major hubs, underscoring the central role of dysregulated immunity in the KIF11 related DEGs. Subsequent functional experiments will be performed to further test the role of KIF11 in modulating these inflammatory pathways. Furthermore, we performed a hierarchical clustering analysis of the enrichment terms based on Jaccard’s similarity index to reduce redundancy and identify distinct functional modules. The resulting cluster tree (Fig 2C) effectively grouped the highly similar GO terms and KEGG pathways into several coherent clusters, including immune receptor activity, leukocyte cell-cell adhesion, signaling receptor activator activity. This clustering reinforces the notion that KIF11’s role in RA is intricately linked to multiple, coordinated aspects of the aberrant immune response. This clustering analysis, based on genes differentially expressed in RA synovium, groups immune and inflammatory pathways into coherent modules. KIF11 is a member of this dysregulated gene set, and its potential involvement in these coordinated immune processes is further investigated through functional experiments in subsequent sections.

**Fig 3:**
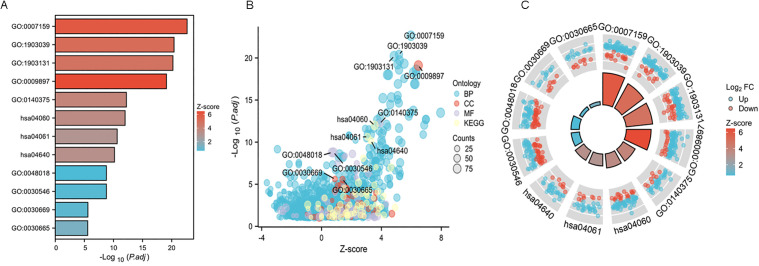
Combined GO/KEGG and logFC enrichment analysis. The enrichment analysis results were visualized using the ggplot2 package (version 3.4.4). **A.** Bar plot. **B.** Bubble plot. **C.** Circle plot.

Moreover, this analysis was uniquely combined with the directional information of gene expression changes (logFC) to provide a more comprehensive functional profile. The results were visualized through multiple complementary graphical representations. The bar plot (Fig 3A) provided a clear ranking of the most significantly enriched terms, effectively highlighting the top pathways based on their Z-scores. Moreover, a bubble plot (Fig 3B) and a circle plot (Fig 3C) were generated to give a more sophisticated overview of the interrelationships between the enriched terms and their association with the gene expression dynamics. This multi-faceted enrichment analysis consistently points towards a state of heightened immune activation in RA, with KIF11 potentially operating within a network of genes that are collectively upregulated to drive pro-inflammatory pathways.

### GSEA substantiates the activation of pro-Inflammatory pathways in RA

To complement the conventional enrichment analysis and gain a systems-level understanding of the transcriptional landscape, we performed GSEA on the 1350 overlapping DEGs. GSEA evaluates the distribution of genes across predefined biological pathways without relying on an arbitrary significance cutoff, thereby capturing more subtle but coordinated expression changes. All enriched gene sets met a significance threshold of an adjusted p-value < 0.05.

**Fig 4:**
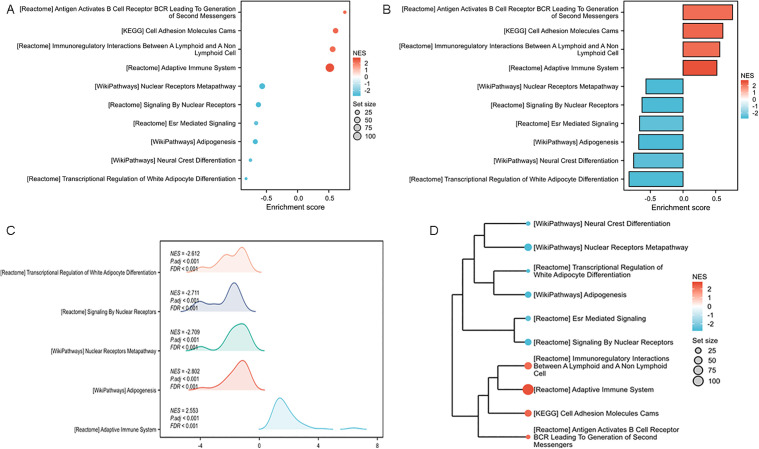
GSEA analysis for 1350 overlapping DEGs in rheumatoid arthritis datasets of GSE55235 and GSE55457. The results of the enrichment analysis and GSEA were visualized using the ggplot2 package *via* bubble plot **(A)**, bar plot **(B)**, and ridge plot **(C)**, with an adjusted p-value < 0.05 **(D)**. The pairwise similarity of enriched terms was calculated using the Jaccard similarity index (JC), followed by cluster analysis of the results using hclust. The clustering outcome was then visualized using the ggplot2 package.

The bubble plot (Fig 4A) and bar plot (Fig 4B) provide a consolidated overview of the most significantly enriched pathways. Both visualizations consistently highlighted a pronounced upregulation of immune-related processes. Key pathways such as “adaptive immune system” and “cell adhesion molecules cams” were prominently ranked at the top, reinforcing the central role of adaptive and innate immune responses in RA pathogenesis.

To visually capture the core enrichment signal within each gene set, we generated a mountain plot (Fig 4C). This visualization clearly demonstrates a strong positive enrichment score (ES) for the aforementioned pathways, indicated by the pronounced peak on the left side of each plot. Moreover, we performed hierarchical clustering based on Jaccard’s similarity index (Fig 4D) and the dendrogram effectively grouped the pathways into distinct functional modules. One major cluster encompassed signaling pathways directly related to adaptive immune system, while another cluster aggregated pathways involved in signaling by nuclear receptors.

### KIF11-correlated Hub genes in RA pathogenesis

**Fig 5:**
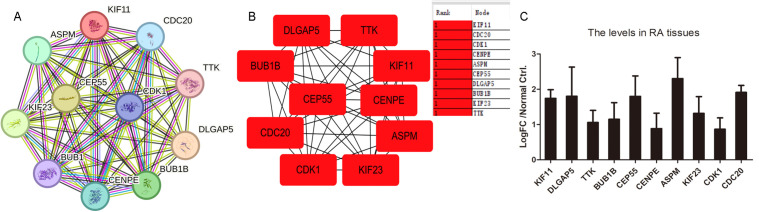
Hub genes correlated with KIF11 and their expression in RA tissues. **A.** Network analyzed using STRING with interaction threshold of 0.40. **B.** Hub genes were predicted using Cytoscape. Top 10 nodes were ranked using MCC. First-stage nodes are shown here. **C.** The expression of top10 hub genes in RA tissues. Log FC values were shown in histogram in five datasets including GSE55457, GSE55235, GSE2053, GSE12021, and GSE1919.

To elucidate the core gene network centered on KIF11 in RA, we constructed a PPI network using the STRING database with a confidence threshold of 0.40 (Fig 5A). We subsequently imported this PPI network into Cytoscape and employed the MCC algorithm to identify the most critical hub nodes. This analysis robustly identified the top 10 hub genes (Fig 5B), which included *KIF11* itself, alongside other key players. The prominence of *KIF11* as a first-stage node within this network strongly implies its pivotal regulatory role in the RA-related gene module.

To investigate the clinical relevance of these computational predictions, we systematically examined the expression patterns of the top 10 hub genes across five independent RA transcriptomic datasets (GSE55457, GSE55235, GSE2053, GSE12021, and GSE1919). The consolidated log FC values, presented in the histogram (Fig 5C), yielded a consistent and compelling finding: the majority of these KIF11-correlated hub genes were significantly upregulated in RA tissues compared to controls. This consistent overexpression across multiple cohorts not only validates our network analysis but also underscores the potential collective contribution of this hub gene network to RA pathology.

### Knockdown of KIF11 suppresses the proliferative, migratory, and invasive phenotypes of MH7A cells

**Fig 6:**
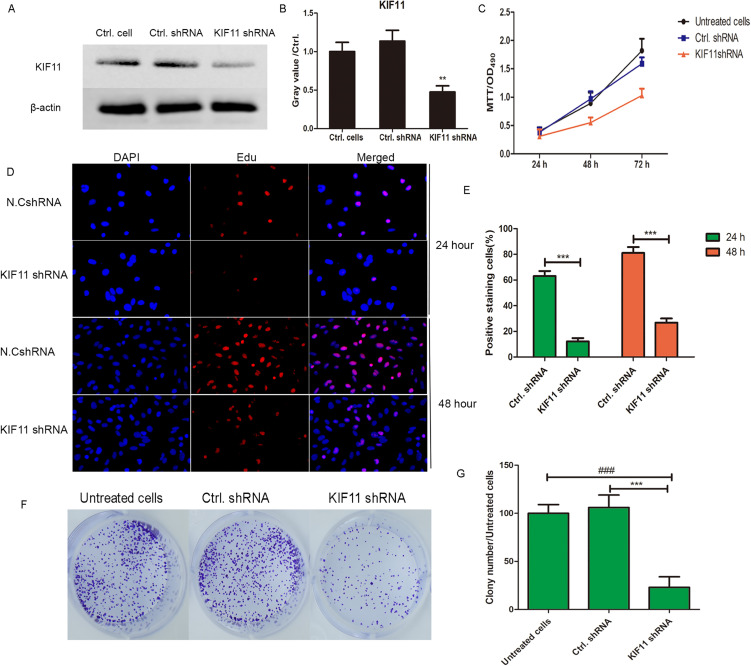
KIF11 knockdown inhibits proliferation, migration and invasion of MH7A cells. **(A)** The expression of KIF11 was detected by western blotting. **(C)**Cell viability was measured by MTT assay at 24, 48, and 72 h post-infection with control shRNA (shCtrl) or KIF11 shRNA (shKIF11) lentivirus. *n* = 3. ***P < 0.001 vs. Ctrl. shRNA group. **(D)** Edu incorporation assay. Fluorescence imaging results at 24 and 48 hours. **E.** The proportion of positively stained cells is displayed in a bar graph, ***P < 0.001. **F.** Colony formation assay. Representative images (E) and quantification (F) of colony formation assay in Ctrl. shRNA- and KIF11 shRNA infected MH7A cells after two-week culture. ***P < 0.001 *vs.* Ctrl.shRNA group, ###p < 0.001 *vs.untreated cells*.

Our preliminary bioinformatics analyses identified KIF11 as one of the hub genes for RA [9]. Meanwhile, given that FLS are key effector cells mediating synovial hyperplasia and joint destruction in RA, and their abnormal proliferation and invasive behavior are closely related to the mitotic function of KIF11, although the transcriptomic data in this study were derived from synovial tissue containing multiple cell types, the central pathological role of FLS led us to prioritize this cell model for functional validation. Therefore, we first investigated the functional role of KIF11 in an RA-derived FLS cell line (MH7A). To directly investigate the functional role of KIF11 in RA fibroblast-like synoviocytes (RA-FLSs), we genetically knocked down KIF11 expression in MH7A cells using a lentivirus-mediated shRNA approach. The efficiency of knockdown was confirmed by western blotting (Fig 6A and 6B). Firstly, the MTT assay revealed that KIF11 knockdown significantly impaired MH7A cell viability in a time-dependent manner, with the suppressive effect becoming more pronounced at 48 and 72 h post-infection (Fig 6C). To further corroborate this finding, we performed an EdU incorporation assay. Consistent with the MTT results, the percentage of EdU-positive cells was dramatically reduced in the shKIF11 group compared to the shCtrl group at both 24 and 48 hours, indicating a substantial inhibition of DNA synthesis and cell proliferation (Fig 6D and 6E, ***P < 0.001). Furthermore, the long-term clonogenic survival ability of MH7A cells was severely compromised upon KIF11 knockdown, as evidenced by a significant reduction in both the number and size of cell colonies formed after two weeks in culture (Fig 6F and 6G).

**Fig 7:**
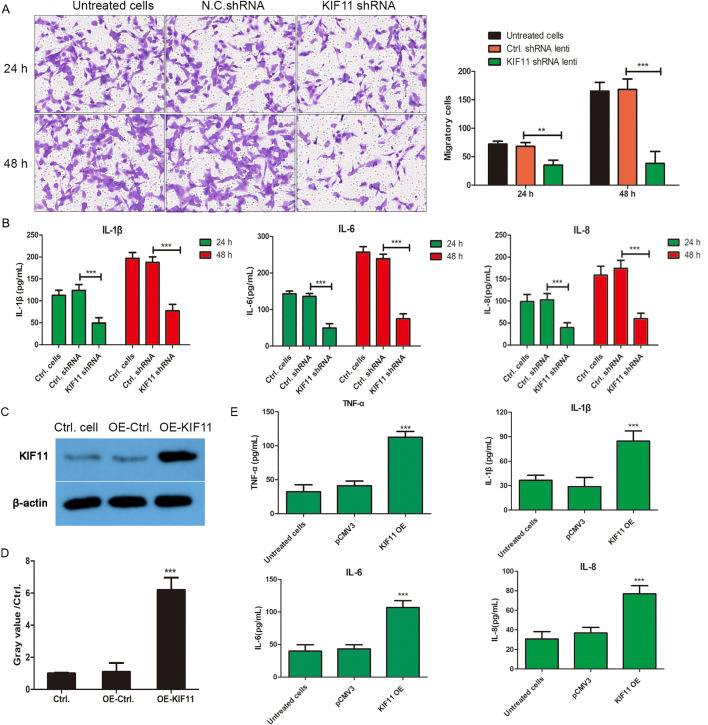
(A) Transwell migration assay of shCtrl- and shKIF11-infected MH7A cells at 24 h and 48 h. Representative images (40 × magnification) and migrated cell quantification (right). Data are mean ± SD (*n* = 3). *P < 0.05, **P < 0.01 *vs.* CtrlshRNA group. (B) The expression of IL-1β, IL-6, and IL-8 was detected by ELISA assay in cell supernatant. ***P < 0.001. (C) Rescue of KIF11 expression in knockdown cells. MH7A cells with stable KIF11 knockdown (shKIF11) were transfected with either a human KIF11 cDNA overexpression plasmid (KIF11-OE) or the empty control vector (Pcmv3 vector). After 24 hours, KIF11 protein expression was analyzed by Western blotting. β-actin served as a loading control. (D) The relative KIF11 protein levels were shown in histogram. Data are presented as mean ± SD (n = 3). ***P < 0.001 vs. the vector-transfected shKIF11 group. (E) Rescue of pro-inflammatory cytokine secretion. The cell culture supernatants from the experiment described in (D) were collected, and the concentrations of TNF-α, IL-1β, IL-6, and IL-8 were measured by ELISA. Data are presented as mean ± SD (n = 3). ***p < 0.001 vs. the control vector-transfected shKIF11 group.

Next, we examined whether KIF11 influences the migratory and invasive capabilities of MH7A cells, which are critical for the pannus formation and joint destruction in RA. The Transwell migration assay demonstrated that the number of migrated cells was significantly lower in the shKIF11 group than in the control group at both 24 and 48 hours (Fig 7A), indicating that KIF11 deficiency attenuates the migratory potential of MH7A cells.

### KIF11 knockdown attenuates the pro-inflammatory function of MH7A cells

Given the pivotal function of FLSs in the inflammatory microenvironment of RA, we also measured the secretion of key pro-inflammatory cytokines. ELISA data revealed that the levels of IL-1β, IL-6, and IL-8 in the cell culture supernatant were markedly decreased following KIF11 knockdown (Fig 7B). This suggests that KIF11 not only drives cellular proliferation and invasion but also contributes to the inflammatory activation of RA-FLSs. However, KIF11 knockdown cannot fully exclude potential off-target effects, we have performed functional rescue experiments in MH7A cells with stable KIF11 knockdown. Specifically, we transfected the knockdown cells with a human KIF11 cDNA ORF Clone overexpression plasmid or the empty control vector (pCMV3-untagged). As shown in the Fig 7C, western blot analysis confirmed that re-expression of KIF11 in the knockdown cells restored KIF11 protein levels to a point significantly higher than both untreated cells and cells transfected with the control vector (***P < 0.001, compared with control plasmid transfected cells, Fig 7D). Concurrently, we measured the secretion of key pro-inflammatory cytokines. The results, presented in the Fig 7E, demonstrated that re-expression of KIF11 effectively reversed the suppression of inflammatory cytokine secretion induced by KIF11 knockdown. Specifically, the levels of TNF-α, IL-1β, IL-6 and IL-8 in the cell culture supernatant were significantly upregulated upon KIF11 overexpression compared to the control vector-transfected knockdown cells.

### KIF11 regulates the NF-κB signaling pathway in RA-FLS

**Fig 8:**
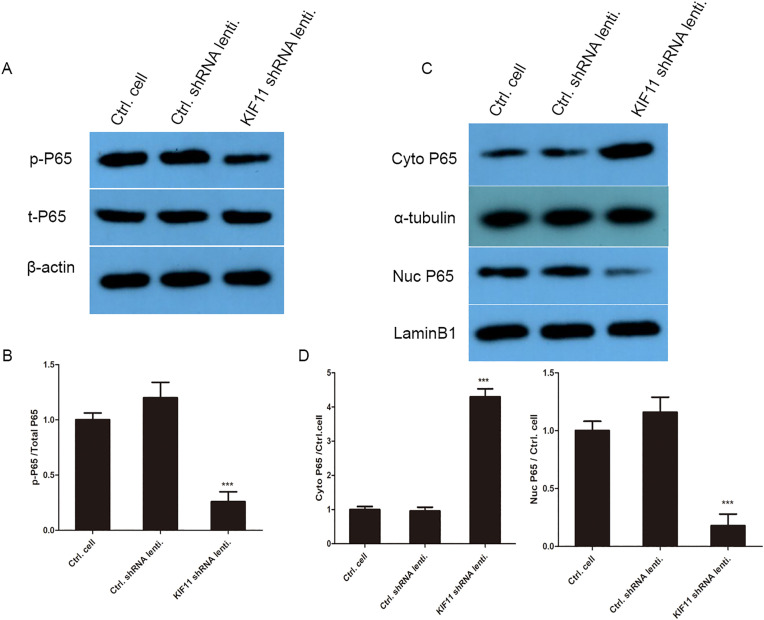
KIF11 regulates the NF-κB signaling pathway in MH7A cells. **(A)** KIF11 knockdown inhibited NF-κB p65 phosphorylation. MH7A cells were infected with control shRNA (shCtrl) or KIF11 shRNA (shKIF11) lentivirus for 24 hours. Whole-cell lysates were subjected to Western blotting using antibodies against phospho-p65 (p-p65), total p65, and KIF11. β-actin served as a loading control. **(B)** The histogram shows the relative ratio of p-p65 to total p65. Data are presented as mean ± SD (n = 3). ***P < 0.001 vs. shCtrl group. **(C)** KIF11 knockdown impaired nuclear translocation of NF-κB p65. Cytoplasmic and nuclear fractions were extracted from shCtrl- and shKIF11-infected MH7A cells. The distribution of p65 in the cytoplasm (Cyto) and nucleus (Nuc) was analyzed by Western blotting. Lamin B1 and α-tubulin were used as markers for nuclear and cytoplasmic fractions, respectively. **(D)**The relative abundance of p65 in the nuclear and cytoplasmic fractions were shown in histograms, normalized to their respective loading controls. Data are presented as mean ± SD (n = 3). ***P < 0.001 vs. shCtrl group.

To explore the molecular mechanism by which KIF11 modulates the inflammatory phenotype of RA-FLS, we first performed GSEA on the overlapping DEGs associated with KIF11. The analysis revealed a significant enrichment of genes involved in the “TNFR2 Non-Canonical NF-κB Pathway” (REACTOME_TNFR2_NON_CANONICAL_NF_KB_PATHWAY, NES = 1.97, p.adj < 0.05), suggesting a potential link between KIF11 and NF-κB signaling, a central regulator of inflammation. To experimentally validate this bioinformatic prediction, we examined the activation status of the NF-κB pathway in MH7A cells following KIF11 knockdown. Western blot analysis showed that phosphorylation of the NF-κB subunit p65 was markedly reduced in shKIF11-infected cells compared to control cells (Fig 8A). Furthermore, to assess the functional consequence of this reduced phosphorylation, we performed subcellular fractionation. The results demonstrated that KIF11 knockdown significantly impaired the nuclear translocation of p65. In control cells stimulated under pro-inflammatory conditions, p65 robustly accumulated in the nucleus. In contrast, this nuclear accumulation was attenuated in KIF11-deficient cells, with a corresponding higher retention of p65 in the cytoplasm (Fig 8B). These data indicate that KIF11 is required for the activation and nuclear translocation of NF-κB in RA-FLS, providing a mechanistic basis for the observed downregulation of NF-κB downstream targets, such as IL-1β and IL-6.

### Knockdown of KIF11 attenuates the M1 macrophage activation marker CD86 and CD80

**Fig 9:**
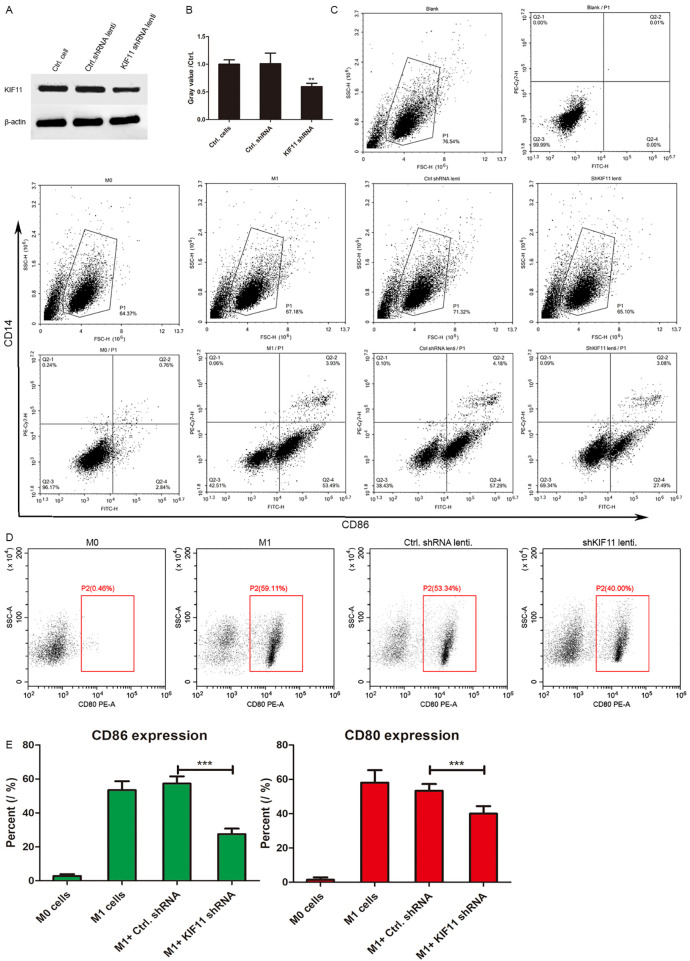
Knockdown of KIF11 in THP-1-derived M1 macrophages suppresses the activation and secretion of inflammatory factors of M1 macrophages. **A.** KIF11 was knock down in M1 macrophages for 24 **h. B.** The histogram of gray value was shown IN Ctrl. shRNA or KIF11 shRNA lentiviruses infected M1 macrophages. **P < 0.01. Interference with KIF11 in M1 macrophages decreased the expression of CD86. The intensity of surface molecules CD14 and CD86 (C) and CD86(D) were determined by flow cytometry in THP-1-derived M0 and M1 cells, shKIF11 lentivirus or ctrl. shRNA lentivirus infected M1 macrophages. **E.** The histogram of CD86 and CD80 expression was shown. ***P < 0.001.

To directly investigate the functional role of KIF11 in macrophage-driven inflammation, we employed a lentivirus-mediated knockdown approach in THP-1-derived M1 macrophages. First, we confirmed the efficiency of KIF11 knockdown at the protein level. Immunoblotting analysis revealed a significant reduction of KIF11 expression in cells transduced with KIF11-specific shRNA (shKIF11) compared to those transduced with a control shRNA (shCtrl) (Fig 9A). Densitometric quantification of the protein bands confirmed a highly efficient knockdown (Fig 9B, **P < 0.01).

We next assessed the impact of KIF11 deficiency on the surface expression of CD86 and CD80, the classical markers for pro-inflammatory M1 macrophages. Flow cytometry analysis demonstrated that while M1 polarization markedly upregulated CD86 expression compared to M0 macrophages, genetic knockdown of KIF11 significantly attenuated the increase of CD86 and CD80 (Fig 9C&9D). Quantitative analysis of the percent of CD86 and CD80 positive cells confirmed a substantial decrease in M1 macrophages with KIF11 depletion, compared with M1 macrophages transfected with control shRNA (Fig 9E, ***P < 0.001), indicating that KIF11 is required for the full activation of the M1 phenotype.

### Knockdown of KIF11 suppresses the expression and secretion of pivotal inflammatory factors

**Fig 10:**
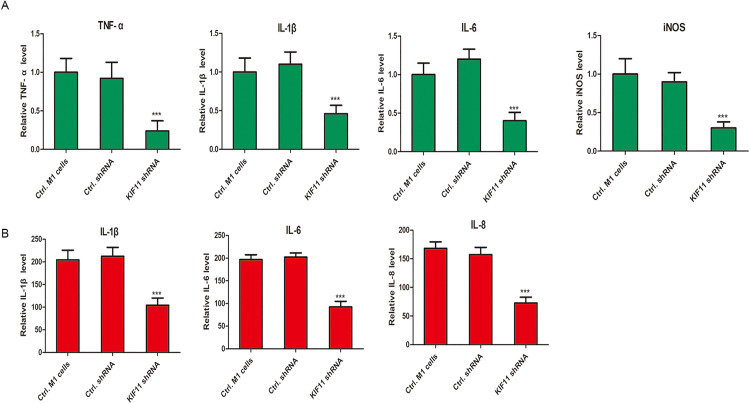
Knockdown of KIF11 in THP-1-derived M1 macrophages suppresses the secretion of inflammatory factors by M1 macrophages. **A.** QPCR assay was performed to test the relative expression of IL-1β, IL-6, iNOS and TNF-*α* in shKIF11 lentivirus infected M1 macrophages and control lentivirus infected M1 macrophages. ***P < 0.001. **B.** ELISA assay. THP-1-derived M1 macrophages were infected with shKIF11 lentivirus or negative control lentivirus for 48 **h.** The concentration of IL-1β, IL-6 and TNF-*α* in cell culture supernatant were tested in shKIF11 lentivirus -infected M1 cells or negative control lentivirus-infected M1 cells. ***P < 0.001, compared with the negative control group.

Given the critical role of cytokines and inflammatory mediators in M1 macrophage function, we examined the transcriptional levels of key pro-inflammatory factors. Quantitative PCR (qPCR) analysis showed that the mRNA expression of IL-1β, IL-6, iNOS, and TNF-α was significantly downregulated in shKIF11-treated M1 macrophages compared to the shCtrl group (Fig 10A, ***P < 0.001). To determine whether this transcriptional suppression translated to a reduction in protein secretion, we measured the cytokine levels in the cell culture supernatant by enzyme-linked immunosorbent assay (ELISA). Consistent with the qPCR data, the secretion of IL-1β, IL-6, and TNF-α was profoundly suppressed in the shKIF11 group compared to the control (Fig 10B, ***P < 0.001).

## Discussion

RA substantially impairs patient quality of life. The intricate pathogenesis of RA involves a complex interplay of immune cells, proinflammatory cytokines, and aberrant signaling pathways that drive synovial hyperplasia and tissue damage [16,17]. Despite advances in therapeutic options, including biologics targeting key inflammatory mediators, a subset of patients remains refractory to treatment, and high relapse rates persist. These clinical challenges underscore the necessity for deeper mechanistic insights into RA pathophysiology, especially regarding the molecular regulators orchestrating synovial fibroblast and macrophage pathogenic behaviors [18]. Elucidating novel molecular contributors beyond classical inflammatory pathways is imperative to identify innovative targets that could improve disease management and patient outcomes.

The present study systematically investigates the role of KIF11 in RA pathogenesis by integrating multi-omics analyses with functional validation. By leveraging transcriptomic data from multiple RA synovial tissue cohorts and corroborating findings in clinical samples and in vitro models, the study reveals a significant upregulation of KIF11 associated with inflammatory activation of fibroblast-like synoviocytes and macrophages. Functional perturbation experiments demonstrate that KIF11 knockdown attenuates cellular proliferation, migration, and inflammatory cytokine production, implicating this motor protein as a pivotal modulator of synovial inflammation and tissue invasion. These findings pave the way for a detailed discussion on the mechanistic significance of KIF11 in RA and its potential as a novel therapeutic target.

Our previous research identified kinesin family member 11 (KIF11) as one of the ten hub genes in the pathogenesis of rheumatoid arthritis (RA) [9]. In the present study, the pronounced upregulation of KIF11 in RA synovial tissue, as well as in MH7A cells following pro-inflammatory cytokine stimulation, highlights a pivotal molecular alteration in the pathogenesis of RA. Prior systematic analyses integrating multiple GEO datasets have identified KIF11 as a robustly upregulated hub gene in RA synovia, with both transcript and protein levels significantly exceeding those in healthy controls. This finding aligns with multi-cohort biomarker discovery studies that consistently place KIF11 among top diagnostic candidates, where its expression correlates strongly with immune cell infiltration, particularly T cells and macrophages [19]. Mechanistically, the regulation of KIF11 by inflammatory mediators such as TNF-α and IL-1β suggests direct transcriptional or post-transcriptional modulation, potentially through canonical inflammatory pathways including NF-κB and JAK/STAT cascades, which are known to orchestrate gene expression programs in chronic inflammatory environments [17]. It is noteworthy that the enhanced KIF11 expression observed in inflammatory and hyperproliferative contexts is not restricted to RA but extends to various cancers and autoimmune diseases, where KIF11 often functions as a cell cycle regulator and is linked to disease aggressiveness or progression [20,21]. However, the direct demonstration of inflammation-induced KIF11 upregulation in FLS models underscores a unique intersection between immunological signaling and cell cycle control that may be particularly relevant to synovial hyperplasia in RA. In summary, the convergence of cytokine regulated KIF11 expression in RA synovium reveals a complex molecular circuitry integrating inflammatory stress with pathological cell cycle activation.

Moreover, the comprehensive functional enrichment analyses consistently revealed that KIF11 and its co-expressed gene networks are intensively involved in immune and inflammatory processes [22]. Multiple bioinformatics studies of RA synovial gene expression have identified KIF11-related modules that are highly enriched in leukocyte adhesion, monocyte differentiation, lymphocyte activation, and cytokine receptor signaling pathways, underscoring the gene’s centrality within immune regulatory circuits [3]. These findings are corroborated by similar network-based analyses in other immune-mediated and proliferative diseases, where KIF11-associated genes cluster within cell cycle, DNA replication, and immune cell communication pathways [23,24]. Furthermore, pan-cancer studies demonstrate that KIF11’s interaction and co-expression networks are not only integral to cell cycle progression but also to antigen processing, immune checkpoint regulation, and the orchestration of the tumor microenvironment [11]. Our findings extend this paradigm to RA, where we discovered that knockdown of KIF11 attenuated M1 macrophage activation and the secretion of pivotal cytokines including IL-1β, IL-6, and TNF-α. The clustering of KIF11 within immune-centric gene networks in our RA bioinformatic analysis supports a model in which this kinesin directly modulates immune cell dynamics within the inflamed synovium.

Based on these findings, we propose a novel, self-amplifying loop that drives RA pathogenesis. In this model, initial inflammatory stimuli within the joint promote synovial cell proliferation and, critically, induce the upregulation of KIF11 in these cells. The elevated KIF11 expression, in turn, acts as a critical regulator that fosters the polarization of macrophages towards a pro-inflammatory M1 phenotype. Subsequently, these activated M1 macrophages secrete copious amounts of IL-1β, IL-6, and TNF-α. These cytokines further fuel the inflammatory milieu, leading to sustained synovial hyperplasia and additional rounds of KIF11 upregulation, thereby creating a positive feedback cycle that perpetuates and exacerbates disease progression.

Functionally, targeted silencing of KIF11 in MH7A fibroblast-like synoviocytes (FLS) has been shown to markedly attenuate their proliferative, clonogenic, migratory, and invasive capacities, implicating KIF11 as a master regulator of the pathological behaviors that underpin synovial hyperplasia and joint destruction in RA. Studies in multiple cancer models corroborate this observation, demonstrating that KIF11 loss-of-function induces cell cycle arrest, impairs DNA synthesis, and reduces tumorigenic potential both in vitro and in vivo [20,25]. Importantly, in gallbladder cancer and hepatocellular carcinoma, KIF11 has been mechanistically linked to the ERBB2/PI3K/AKT and Wnt/β-catenin pathways, with its activity modulated by epigenetic factors and protein stability regulators such as USP1 [25–27]. In the context of RA, the parallels between synovial FLS and malignant cells—both exhibiting unchecked proliferation, resistance to apoptosis, and invasive growth—highlight the relevance of these mechanistic insights. Comparative analyses further reveal that KIF11’s regulation of cytoskeletal dynamics and microtubule-based processes is intimately intertwined with the activity of canonical FLS effector molecules such as matrix metalloproteinases (MMPs), integrins, and cyclins, yet KIF11 appears to exert broader control over cell cycle and migratory programs [28,29]. Thus, the suppression of FLS pathological traits upon KIF11 depletion suggests that this kinesin integrates multiple oncogenic and inflammatory cues to drive tissue remodeling in RA.

Interestingly, KIF11 knockdown in MH7A cells exhibited a dynamic inhibitory pattern on DNA synthesis: while EdU incorporation was profoundly suppressed at 24 h, a partial recovery was observed at 48 h, albeit remaining significantly lower than controls (Fig 6D-E). This suggests that KIF11 depletion induces a transient rather than permanent cell cycle arrest. The partial recovery may reflect adaptive cellular responses to KIF11 deficiency, such as compensatory upregulation of alternative mitotic kinesins, activation of bypass cell cycle checkpoint pathways, or residual KIF11 expression in a subset of cells that gradually re-enter the cell cycle. Importantly, however, the long-term colony formation assay revealed that KIF11 knockdown nearly abolished the ability of MH7A cells to form colonies over a two-week culture period (Fig 6F-G), demonstrating a sustained and fundamental impairment of FLS proliferative and self-renewal capacity. These seemingly disparate findings collectively illuminate a critical biological insight: while KIF11 is a key regulator of FLS hyperplasia, achieving optimal therapeutic suppression of synovial proliferation in RA may require sustained and complete KIF11 inhibition. Future therapeutic strategies should explore whether combining KIF11 inhibitors with agents targeting alternative cell cycle pathways or survival mechanisms could overcome potential compensatory responses, thereby achieving more profound and durable suppression of synovial hyperplasia in RA.

Beyond its role in FLS biology, KIF11 depletion significantly reduces the secretion of key pro-inflammatory cytokines—including IL-1β, IL-6, and IL-8—and inhibits the polarization and activation of M1-type macrophages, as indicated by decreased expression of CD86, iNOS, and TNF-α. These observations resonate with pan-cancer and inflammatory disease studies, where KIF11 is implicated in modulating immune cell infiltration, cytokine signaling, and the broader inflammatory milieu [11,30]. In osteoclasts, for example, KIF11 has been shown to regulate the NF-κB pathway via mTORC1, thereby influencing differentiation and inflammatory osteolysis [31]. Moreover, investigations into FLS-macrophage interactions in RA and other autoimmune contexts emphasize the importance of reciprocal cytokine exchange and contact-dependent signaling for sustaining chronic inflammation and tissue destruction [4]. Notably, KIF11’s impact on immune cell polarization and cytokine production appears to be both cell-intrinsic and extrinsic, affecting not only the effector phenotype of macrophages but also the inflammatory potential of stromal cells. Collectively, these data delineate a dual role for KIF11 in orchestrating both local synovial cell pathology and the broader immune-inflammatory axis within the RA microenvironment.

Despite the robust integration of multi-cohort transcriptomic datasets and the employment of both *in vitro* and *ex vivo* validation strategies, this study is limited by the restricted number and heterogeneity of clinical synovial specimens, which may not fully capture the spectrum of rheumatoid arthritis phenotypes or accommodate inter-individual variability in gene expression. Additionally, the reliance on immortalized MH7A and THP-1-derived macrophage models, while enabling controlled mechanistic interrogation, may not entirely recapitulate the complexity of the in vivo synovial microenvironment or the multifaceted cellular interactions driving RA pathogenesis. Future research should prioritize the validation of KIF11’s functional role in primary macrophages derived from the synovial fluid and peripheral blood of RA patients. Specifically, it will be essential to correlate KIF11 expression levels with disease activity and macrophage activation states in clinical samples, and to directly test whether genetic silencing or pharmacological inhibition of KIF11 can attenuate the pro-inflammatory cytokine production and hyper-activated phenotype characteristic of RA-associated macrophages. These studies are indispensable for confirming the pathophysiological relevance of our findings in immortalized cell models and for critically evaluating the translational feasibility of targeting KIF11 in RA.

The pathogenic centrality of KIF11 in RA synovitis positions it as a compelling therapeutic target. Fortunately, KIF11 has been extensively pursued in oncology, providing a rich repository of clinical data and compounds for potential repurposing [32,33]. Several classes of KIF11 inhibitors have been developed, including ATP-competitive agents like Ispinesib and allosteric inhibitors like Filanesib, which work by disrupting mitotic spindle assembly and arresting proliferating cells [34–36]. Clinical trials, however, reveal a complex picture. In solid tumors, single-agent KIF11 inhibitors have shown limited efficacy, often constrained by dose-limiting hematological toxicities, primarily neutropenia, reflecting the protein’s essential role in rapidly dividing cells [36,37]. More promising activity has been observed in hematological malignancies like multiple myeloma, where Filanesib combined with dexamethasone showed efficacy in specific patient subsets [34]. Common challenges across indications include primary and acquired resistance mechanisms (e.g., compensatory pathways involving other kinesins like KIF15) and a current lack of validated predictive biomarkers to select patients most likely to benefit [37,38].

Translating these lessons to RA requires strategic consideration. The RA synovium shares with tumors a hyperproliferative and invasive phenotype. Our data demonstrate that KIF11 knockdown not only suppresses fibroblast-like synoviocyte proliferation and migration but also attenuates M1 macrophage activation and pro-inflammatory cytokine (e.g., IL-1β, IL-6, TNF-α) production. This dual role suggests that KIF11 inhibition in RA could uniquely target both the “tumor-like” growth of the synovium and the underlying inflammatory drive. The lower incidence of neurotoxicity compared to traditional microtubule disruptors (e.g., taxanes) could be a relative advantage for chronic musculoskeletal conditions [39]. However, the systemic toxicity profile, especially myelosuppression, remains a significant hurdle for chronic autoimmune disease therapy. Therefore, future development for RA should prioritize strategies to maximize local joint exposure while minimizing systemic effects, such as intra-articular delivery systems or the development of soft drugs [40,41]. Furthermore, identifying RA patient subsets with high synovial KIF11 expression or specific pathogenic pathways dependent on KIF11 could enable biomarker-driven clinical trials, increasing the likelihood of success. Exploring rational combinations, for instance with conventional DMARDs or biologic agents targeting complementary pathways, may also enhance efficacy and allow for lower, less toxic doses of a KIF11 inhibitor. In conclusion, while direct repurposing faces challenges, the clinical experience in oncology provides a crucial roadmap. Our study furnishes the mechanistic rationale, and a tailored approach informed by oncology’s lessons could unlock KIF11’s potential as a novel therapeutic strategy for refractory RA.

In summary, our findings define KIF11 as a critical regulator of synovial cell proliferation, migration, and pro-inflammatory activation, with network analyses implicating it at the center of immune-related gene modules in RA. The attenuation of both FLS and macrophage inflammatory phenotypes following KIF11 knockdown highlights its dual functional relevance and positions KIF11 as a potential therapeutic target. Future research should prioritize in vivo validation using primary patient-derived synovial cells and animal models, as well as the exploration of KIF11-targeted interventions, to evaluate translational feasibility and elucidate broader immunopathogenic mechanisms within RA.

## Supporting information

S1 Text The sequences of the primers for real time qPCR. (DOCX)

S1 Fig Raw images. (PDF)

## References

[1] Li Z, Xu M, Li R, Zhu Z, Liu Y, Du Z, et al. Identification of biomarkers associated with synovitis in rheumatoid arthritis by bioinformatics analyses. Biosci Rep. 2020;40(9):BSR20201713. doi: 10.1042/BSR20201713 32840301 PMC7502692

[2] Gao W, Lu J, Yang Z, Li E, Cao Y, Xie L. Mitotic Functions and Characters of KIF11 in Cancers. Biomolecules. 2024;14(4):386. doi: 10.3390/biom14040386 38672404 PMC11047945

[3] Wu Y-K, Liu C-D, Liu C, Wu J, Xie Z-G. Machine learning and weighted gene co-expression network analysis identify a three-gene signature to diagnose rheumatoid arthritis. Front Immunol. 2024;15:1387311. doi: 10.3389/fimmu.2024.1387311 38711508 PMC11070572

[4] Fang Q, Li T, Chen P, Wu Y, Wang T, Mo L, et al. Comparative Analysis on Abnormal Methylome of Differentially Expressed Genes and Disease Pathways in the Immune Cells of RA and SLE. Front Immunol. 2021;12:668007. doi: 10.3389/fimmu.2021.668007 34079550 PMC8165287

[5] Khan B, Qahwaji R, Alfaifi MS, Athar T, Khan A, Mobashir M, et al. Deciphering molecular landscape of breast cancer progression and insights from functional genomics and therapeutic explorations followed by in vitro validation. Sci Rep. 2024;14(1):28794. doi: 10.1038/s41598-024-80455-6 39567714 PMC11579425

[6] Auwul MR, Rahman MR, Gov E, Shahjaman M, Moni MA. Bioinformatics and machine learning approach identifies potential drug targets and pathways in COVID-19. Brief Bioinform. 2021;22(5):bbab120. doi: 10.1093/bib/bbab120 33839760 PMC8083354

[7] Sakuma Y, Hirai S, Yamaguchi M, Idogawa M. Small Cell Lung Carcinoma Cells Depend on KIF11 for Survival. Int J Mol Sci. 2024;25(13):7230. doi: 10.3390/ijms25137230 39000337 PMC11241341

[8] Meißner L, Niese L, Schüring I, Mitra A, Diez S. Human kinesin-5 KIF11 drives the helical motion of anti-parallel and parallel microtubules around each other. EMBO J. 2024;43(7):1244–56. doi: 10.1038/s44318-024-00048-x 38424239 PMC10987665

[9] Ban ZYW, Xu L, Lan X, Xing S, Ye Y, Xu B, et al. Upregulation of hub gene RRM2 promotes progression of rheumatoid arthritis. Journal of Biological Regulators and Homeostatic Agents. 2022;36(3):655–66.

[10] Silva JPN, Silva PMA, Bousbaa H. Kinesin spindle protein (KIF11) in mitosis and cancer. Int J Mol Sci. 2025;26(18). 10.3390/ijms26188975 PMC12469638 41009541

[11] Guo X, Zhou L, Wu Y, Li J. KIF11 as a potential pan-cancer immunological biomarker encompassing the disease staging, prognoses, tumor microenvironment, and therapeutic responses. Oxid Med Cell Longev. 2022;2022:2764940. 36742345 10.1155/2022/2764940 PMC9893523

[12] Ricci A, Carradori S, Cataldi A, Zara S. Eg5 and Diseases: From the Well-Known Role in Cancer to the Less-Known Activity in Noncancerous Pathological Conditions. Biochem Res Int. 2024;2024:3649912. doi: 10.1155/2024/3649912 38939361 PMC11211015

[13] Gaba RC, Groth JV, Parvinian A, Guzman G, Casadaban LC. Gene expression in hepatocellular carcinoma: pilot study of potential transarterial chemoembolization response biomarkers. J Vasc Interv Radiol. 2015;26(5):723–32. doi: 10.1016/j.jvir.2014.12.610 25724086

[14] Ban Z, Li Z, Xing S, Ye Y. IGF2BP3 regulates the expression of RRM2 and promotes the progression of rheumatoid arthritis via RRM2/Akt/MMP-9 pathway. PLoS One. 2024;19(5):e0303593. doi: 10.1371/journal.pone.0303593 38820515 PMC11142689

[15] Li Z, Zhang Y, Li Y, Xing S, Li S, Lyu J, et al. PROSER2 is a poor prognostic biomarker for patients with osteosarcoma and promotes proliferation, migration and invasion of osteosarcoma cells. Exp Ther Med. 2022;24(6):750. doi: 10.3892/etm.2022.11686 36561964 PMC9748638

[16] Anandan M, Narayanan J. Role of T cells and cytokines in the pathogenesis of rheumatoid arthritis. Biochem Biophys Rep. 2025;44:102278. doi: 10.1016/j.bbrep.2025.102278 41127878 PMC12538455

[17] Liu Y, Wang X-Q, Lu J-H, Huang H-W, Qiu Y-H, Peng Y-P. Treg cells mitigate inflammatory responses and symptoms via β2-AR/β-Arr2/ERK signaling in an experimental rheumatoid arthritis. Arthritis Res Ther. 2025;27(1):194. doi: 10.1186/s13075-025-03659-9 41107955 PMC12532925

[18] Georgios I, Fani S, Dmitrios B, David L, Dimitrios D. Immune checkpoint inhibitor-induced inflammatory arthritis vs rheumatoid arthritis: A comparative review. Autoimmun Rev. 2026;25(1):103943. doi: 10.1016/j.autrev.2025.103943 41115565

[19] Xu J, Gong Y, Ding L, Luo Y, Wang R, Ren T. AIM2 as an Immunogenic Cell Death (ICD)-related hub gene in rheumatoid arthritis: identification and functional validation. Clin Rheumatol. 2025;44(12):4865–79. doi: 10.1007/s10067-025-07728-x 41116042

[20] Wei D, Rui B, Qingquan F, Chen C, Ping HY, Xiaoling S, et al. KIF11 promotes cell proliferation via ERBB2/PI3K/AKT signaling pathway in gallbladder cancer. Int J Biol Sci. 2021;17(2):514–26. doi: 10.7150/ijbs.54074 33613109 PMC7893577

[21] Pei Y-Y, Li G-C, Ran J, Wan X-H, Wei F-X, Wang L. Kinesin Family Member 11 Enhances the Self-Renewal Ability of Breast Cancer Cells by Participating in the Wnt/β-Catenin Pathway. J Breast Cancer. 2019;22(4):522–32. doi: 10.4048/jbc.2019.22.e51 31897327 PMC6933027

[22] Zhi X, Zhu H, Sun X, Wang Z, Wang L, Song G. Dysregulation of Different Modes of Programmed Cell Death in Rheumatoid Arthritis Fibroblast-Like Synoviocyte. Int J Rheum Dis. 2025;28(10):e70445. doi: 10.1111/1756-185x.70445 41128076 PMC12548005

[23] Zhu L, Chen C, Kang M, Ma X, Sun X, Xue Y, et al. KIF11 serves as a cell cycle mediator in childhood acute lymphoblastic leukemia. J Cancer Res Clin Oncol. 2023;149(17):15609–22. doi: 10.1007/s00432-023-05240-w 37656243 PMC10620298

[24] Fei H, Chen S, Xu C. Bioinformatics analysis of gene expression profile of serous ovarian carcinomas to screen key genes and pathways. J Ovarian Res. 2020;13(1):82. doi: 10.1186/s13048-020-00680-1 32693821 PMC7374965

[25] Wu Z, Li C, Zhang S, Sun L, Hu J, Qiu B, et al. High interstitial fluid pressure enhances USP1-dependent KIF11 protein stability to promote hepatocellular carcinoma progression. J Transl Med. 2025;23(1):66. doi: 10.1186/s12967-025-06124-y 39810156 PMC11730827

[26] Song P, Lv D, Yang L, Zhou J, Yan X, Liu Z, et al. Di-(2-ethylhexyl) phthalate promotes benign prostatic hyperplasia through KIF11-Wnt/β-catenin signaling pathway. Ecotoxicol Environ Saf. 2024;281:116602. doi: 10.1016/j.ecoenv.2024.116602 38944010

[27] Wu B, Hu C, Kong L. ASPM combined with KIF11 promotes the malignant progression of hepatocellular carcinoma via the Wnt/β-catenin signaling pathway. Exp Ther Med. 2021;22(4):1154. doi: 10.3892/etm.2021.10588 34504599 PMC8393588

[28] Liu X, Ren Y, Qin S, Yang Z. Exploring the mechanism of 6-Methoxydihydrosanguinarine in the treatment of lung adenocarcinoma based on network pharmacology, molecular docking and experimental investigation. BMC Complement Med Ther. 2024;24(1):202. doi: 10.1186/s12906-024-04497-z 38783288 PMC11119275

[29] Arora S, Singh P, Rahmani AH, Almatroodi SA, Dohare R, Syed MA. Unravelling the Role of miR-20b-5p, CCNB1, HMGA2 and E2F7 in Development and Progression of Non-Small Cell Lung Cancer (NSCLC). Biology (Basel). 2020;9(8):201. doi: 10.3390/biology9080201 32752229 PMC7465122

[30] Liu Z, Petinrin OO, Chen N, Toseef M, Liu F, Zhu Z, et al. Identification and evaluation of candidate COVID-19 critical genes and medicinal drugs related to plasma cells. BMC Infect Dis. 2024;24(1):1099. doi: 10.1186/s12879-024-10000-3 39363208 PMC11451256

[31] Miao J, Yao H, Liu J, Huang Z, Shi C, Lu X, et al. Inhibition of KIF11 ameliorates osteoclastogenesis via regulating mTORC1-mediated NF-κB signaling. Biochem Pharmacol. 2023;217:115817. doi: 10.1016/j.bcp.2023.115817 37757917

[32] Yang G, Fu J, Wang J, Ding M. HELLS Knockdown Inhibits the Malignant Progression of Lung Adenocarcinoma Via Blocking Akt/CREB Pathway by Downregulating KIF11. Mol Biotechnol. 2025;67(2):548–61. doi: 10.1007/s12033-024-01066-0 38478260

[33] Gampa G, Kenchappa RS, Mohammad AS, Parrish KE, Kim M, Crish JF, et al. Enhancing Brain Retention of a KIF11 Inhibitor Significantly Improves its Efficacy in a Mouse Model of Glioblastoma. Sci Rep. 2020;10(1):6524. doi: 10.1038/s41598-020-63494-7 32300151 PMC7162859

[34] Garcia-Saez I, Skoufias DA. Eg5 targeting agents: From new anti-mitotic based inhibitor discovery to cancer therapy and resistance. Biochem Pharmacol. 2021;184:114364. doi: 10.1016/j.bcp.2020.114364 33310050

[35] Jungwirth G, Yu T, Cao J, Eddine MA, Moustafa M, Warta R, et al. KIF11 inhibitors filanesib and ispinesib inhibit meningioma growth in vitro and in vivo. Cancer Lett. 2021;506:1–10. doi: 10.1016/j.canlet.2021.02.016 33652084

[36] Cai L, Wang J, Yang Y, Cheng J, Wei Y, Su X, et al. KIF11 promotes AML progression, and its inhibition by SB-743921 suppresses disease advancement through mitotic G2/M phase arrest. Cell Signal. 2025;135:111980. doi: 10.1016/j.cellsig.2025.111980 40659169

[37] Lau TT, Ma HT, Poon RY. Kinesins regulate the heterogeneity in centrosome clustering after whole-genome duplication. Life Sci Alliance. 2024;7(10):e202402670. doi: 10.26508/lsa.202402670 39074902 PMC11287020

[38] Sun R-F, He N, Zhang G-Y, Yu Z-Y, Li L-S, Ma Z-J, et al. Combined Inhibition of KIF11 and KIF15 as an Effective Therapeutic Strategy for Gastric Cancer. Curr Cancer Drug Targets. 2023;23(4):293–306. doi: 10.2174/1568009622666220616122846 35713129

[39] Skinner MW, Simington CJ, López-Jiménez P, Baran KA, Xu J, Dayani Y, et al. Spermatocytes have the capacity to segregate chromosomes despite centriole duplication failure. EMBO Rep. 2024;25(8):3373–405. doi: 10.1038/s44319-024-00187-6 38943004 PMC11316026

[40] Singh E, Osmani RAM, Banerjee R, Abu Lila AS, Moin A, Almansour K, et al. Poly epsilon-Caprolactone Nanoparticles for Sustained Intra-Articular Immune Modulation in Adjuvant-Induced Arthritis Rodent Model. Pharmaceutics. 2022;14(3). 10.3390/pharmaceutics14030519 PMC8953799 35335895

[41] Gomes TF, Gualberto FCM, Perasoli FB, Andrade FP, Moura SAL, Da Silva GR. Intra-articular leflunomide-loaded poly(epsilon-caprolactone) implants to treat synovitis in rheumatoid arthritis. Pharmazie. 2019;74(4):212–20. 30940304 10.1691/ph.2019.8223

[42] Mora A, Donaldson IM. iRefR: an R package to manipulate the iRefIndex consolidated protein interaction database. BMC Bioinformatics. 2011;12:455. doi: 10.1186/1471-2105-12-455 22115179 PMC3282787

