# Supplementary material for: KIF11 promotes rheumatoid arthritis pathogenesis by activating M1 macrophage polarization and promoting inflammatory cytokine secretion
Source: PLoS One. 2026 May 13;21(5):e0347313. doi: 10.1371/journal.pone.0347313 (PMC13170830; doi:10.1371/journal.pone.0347313)
Supplement: S1 Text — (DOCX) [file pone.0347313.s001.docx]

S1 text

The sequences of the primers for real time qPCR

TNF-α:

5'-CATCTTCTCAAAATTCGAGTGACAA-3' and 5'-TGGGAGTAGACAAGGTACAACCC-3';

IL-1β:

5'-TCATTGTGGCTGTGGAGAAG-3' and 5'-AGGCCACAGGTATTTTGTCG-3';

IL-6:

5'-ATCCAGTTGCCTTCTTCTTGGGACTGA-3' and 5'-TAAGCCTCCGACTTGTGAAGTGGT-3';

iNOS:

5'-CTGCAGCACTTGGATCAGGAACCTG-3' and 5'-GGAGTAGCCTGTGTGCACCTGGAA-3';

β-actin:

5'-AGAGGGAAATCGTGCGTGAC-3' and 5'-CAATAGTGATGACCTGGCCGT-3'.
