# Supplementary material for: KIF11 promotes rheumatoid arthritis pathogenesis by activating M1 macrophage polarization and promoting inflammatory cytokine secretion
Source: PLoS One. 2026 May 13;21(5):e0347313. doi: 10.1371/journal.pone.0347313 (PMC13170830; doi:10.1371/journal.pone.0347313)
Supplement: S1 Fig — (PDF) [file pone.0347313.s002.pdf]

S2 Fig.

Raw images

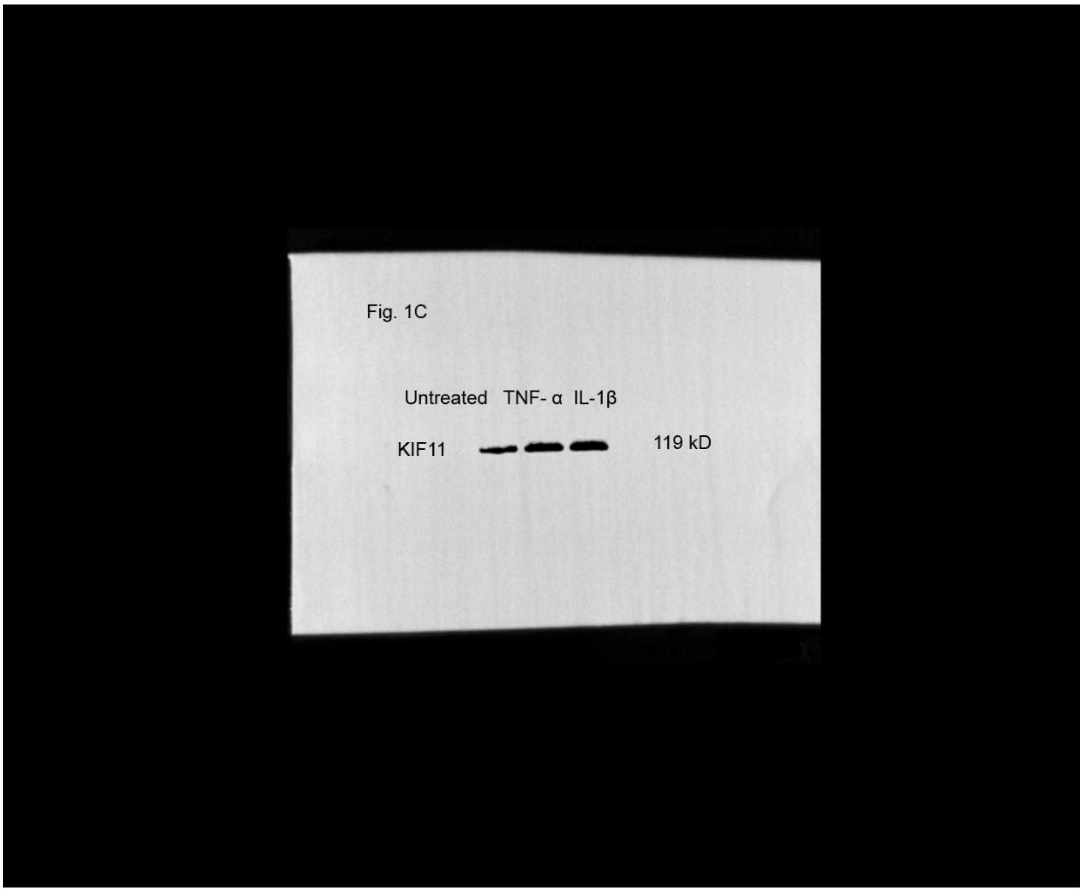

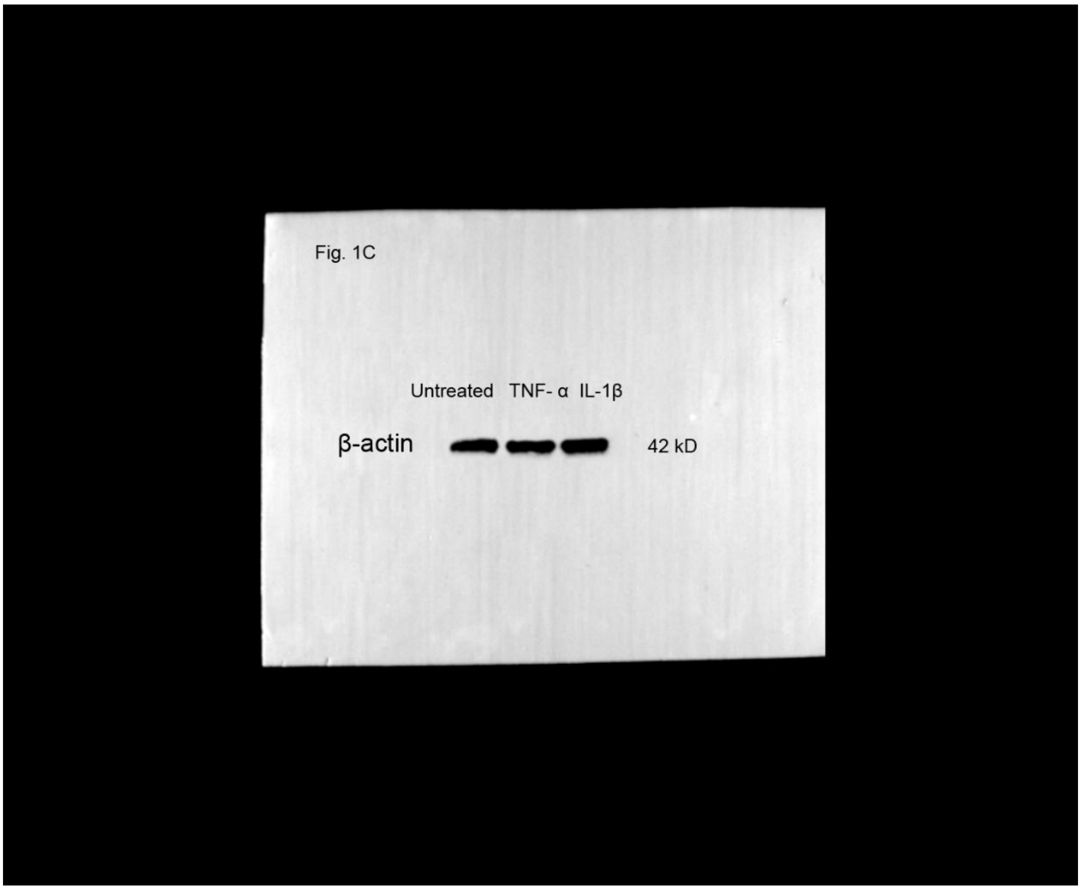
Fig. 1C
Untreated TNF- α IL-1β
β-actin
42 kD

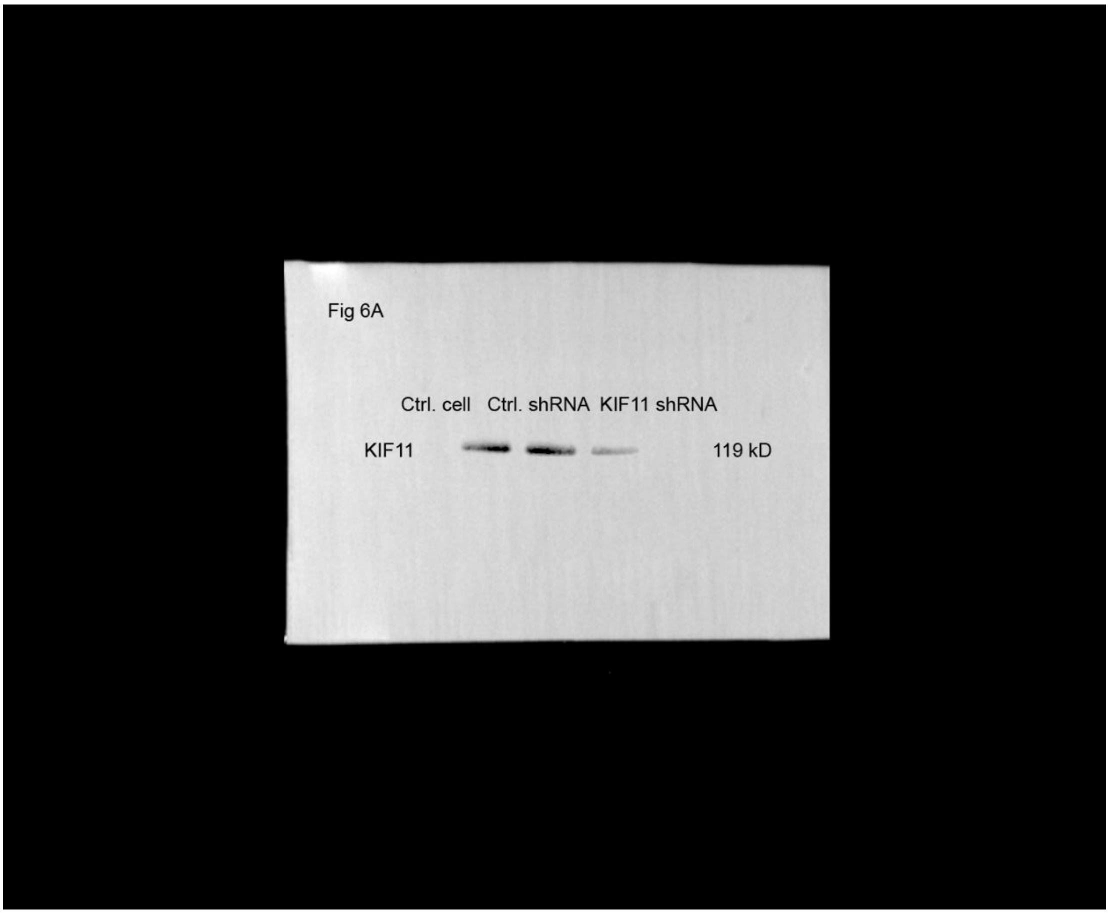
Fig 6A
Ctrl. cell Ctrl. shRNA KIF11 shRNA
KIF11
119 kD

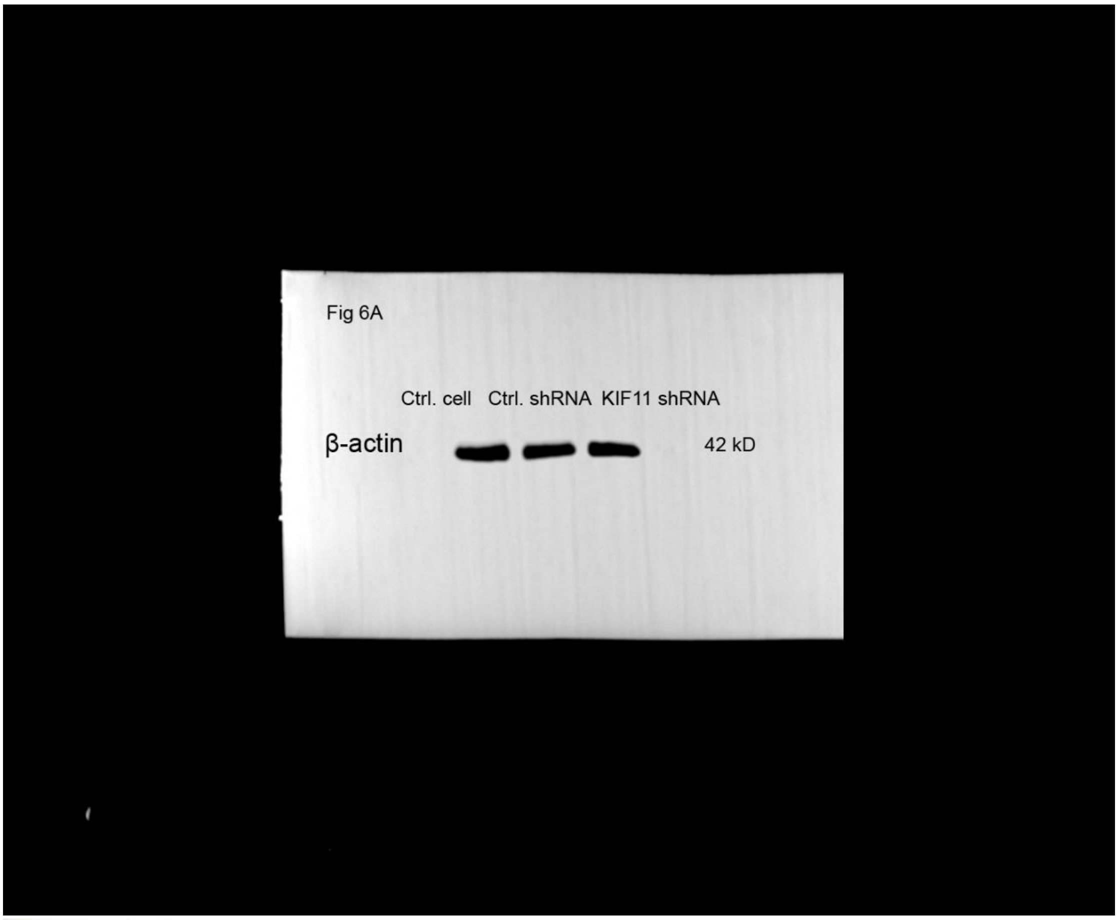

Fig 6A
Ctrl. cell
Ctrl. shRNA
KIF11 shRNA
β-actin
42 kD

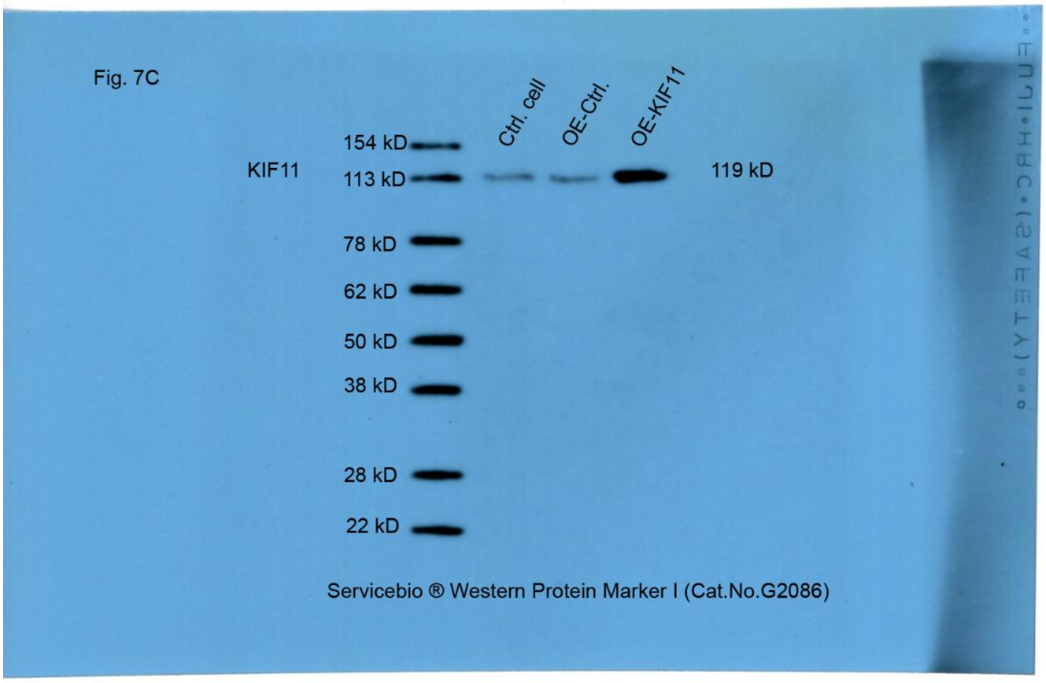

Fig. 7C
Ctrl. cell
OE-Ctrl.
OE-KIF11
KIF11
154 kD
113 kD
78 kD
62 kD
50 kD
38 kD
28 kD
22 kD
119 kD
Servicebio ® Western Protein Marker I (Cat.No.G2086)

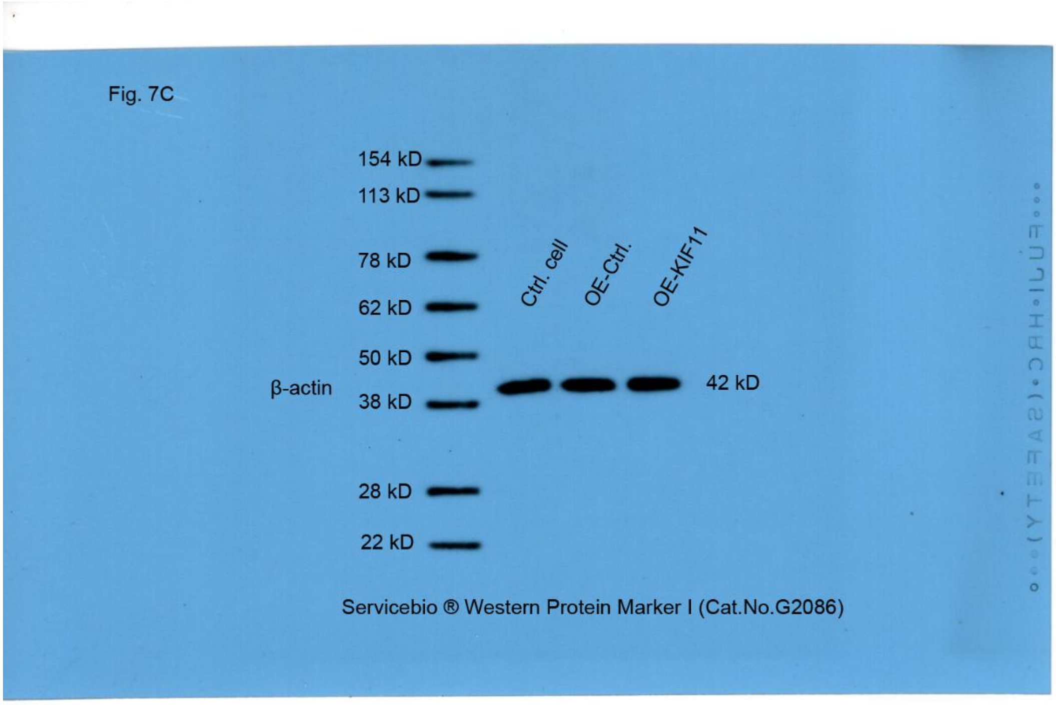
Fig. 7C
154 kD
113 kD
78 kD
62 kD
50 kD
38 kD
28 kD
22 kD
Ctrl. cell
OE-Ctrl.
OE-KIF11
β-actin
42 kD
Servicebio ® Western Protein Marker I (Cat.No.G2086)

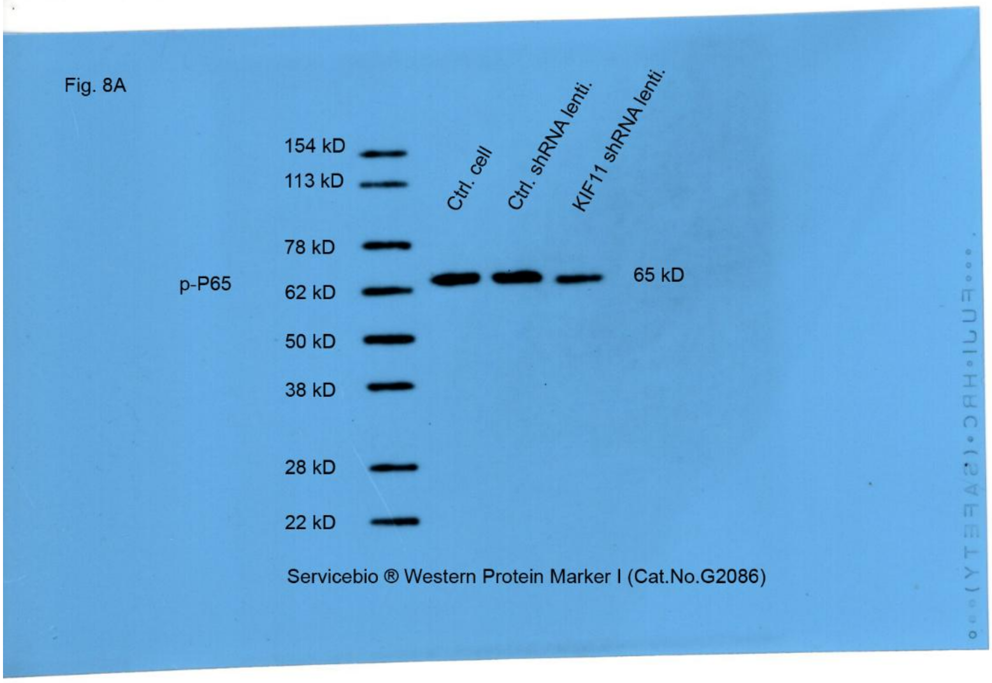
Fig. 8A
154 kD
113 kD
78 kD
62 kD
50 kD
38 kD
28 kD
22 kD
Ctrl. cell
Ctrl. shRNA lenti.
KIF11 shRNA lenti.
p-P65
65 kD
Servicebio ® Western Protein Marker I (Cat.No.G2086)

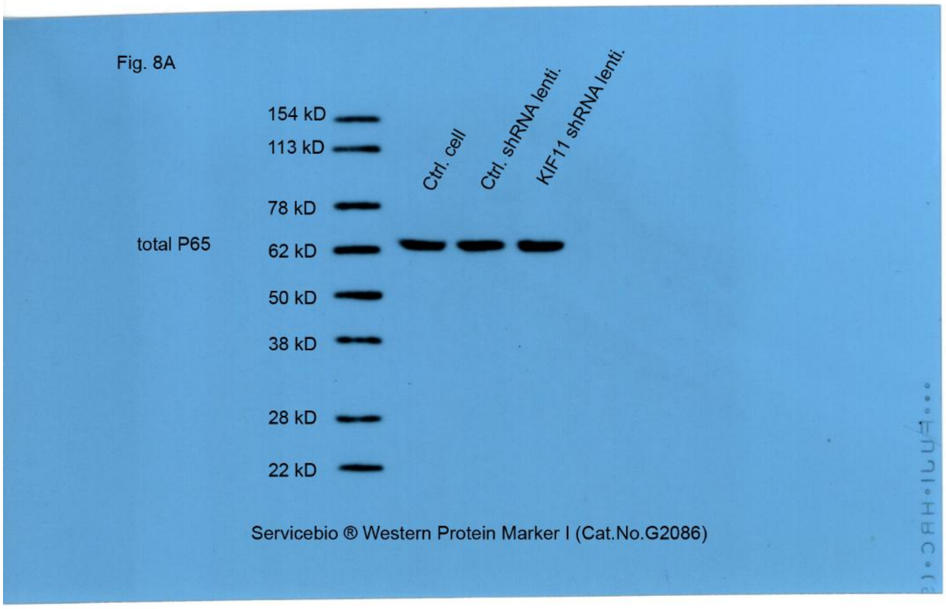
Fig. 8A
154 kD
113 kD
78 kD
62 kD
50 kD
38 kD
28 kD
22 kD
total P65
Ctrl. cell
Ctrl. shRNA lenti.
KIF11 shRNA lenti.
Servicebio ® Western Protein Marker I (Cat.No.G2086)

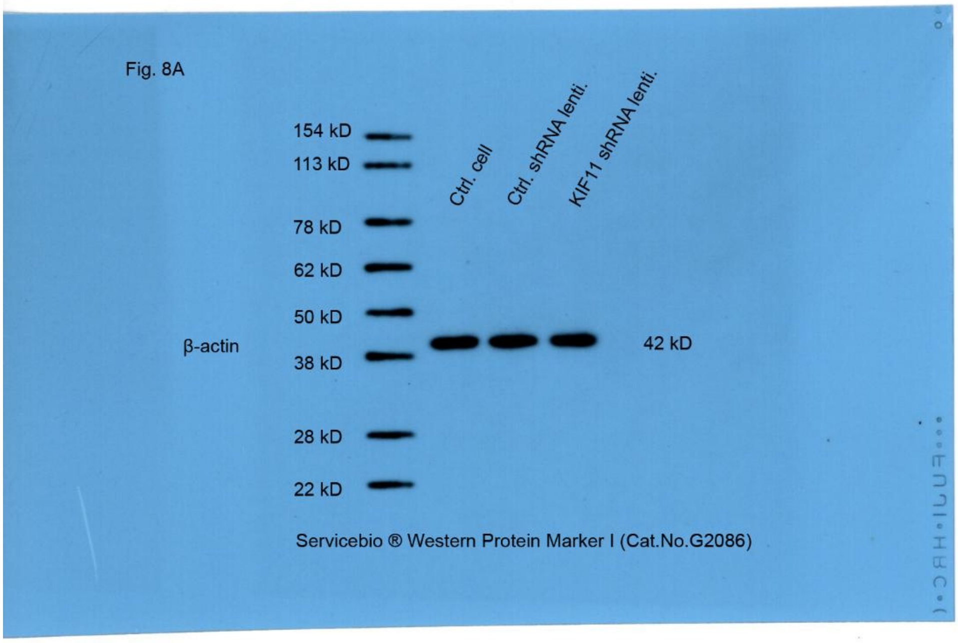
Fig. 8A
154 kD
113 kD
78 kD
62 kD
50 kD
38 kD
28 kD
22 kD
β-actin
Ctrl. cell
Ctrl. shRNA lenti.
KIF11 shRNA lenti.
42 kD
Servicebio ® Western Protein Marker I (Cat.No.G2086)

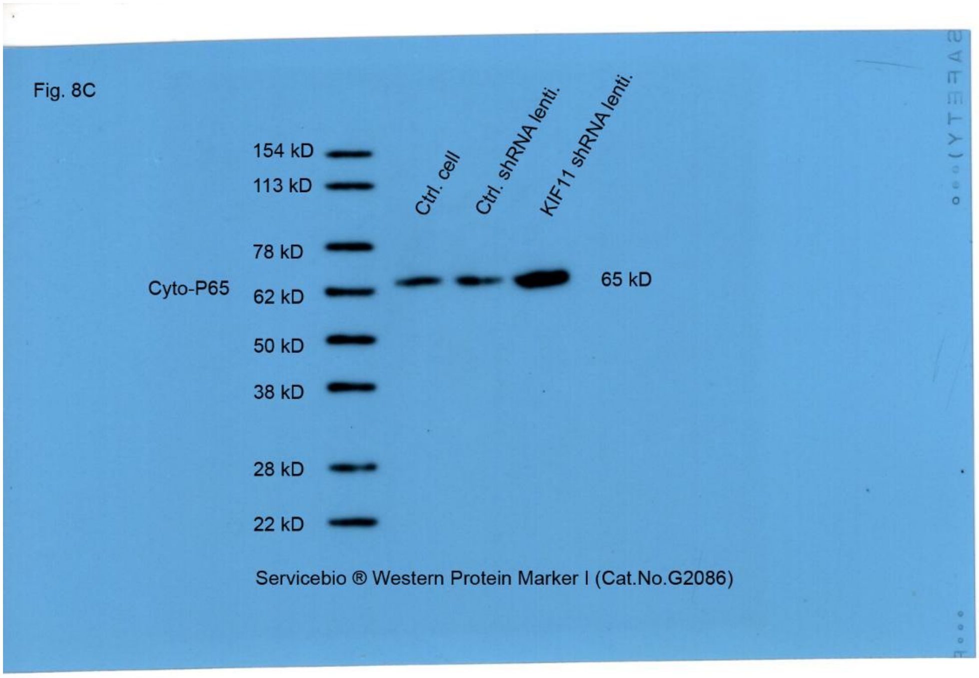
Fig. 8C
154 kD
113 kD
78 kD
62 kD
50 kD
38 kD
28 kD
22 kD
Ctrl. cell
Ctrl. shRNA lenti.
KIF11 shRNA lenti.
Cyto-P65
65 kD
Servicebio ® Western Protein Marker I (Cat.No.G2086)

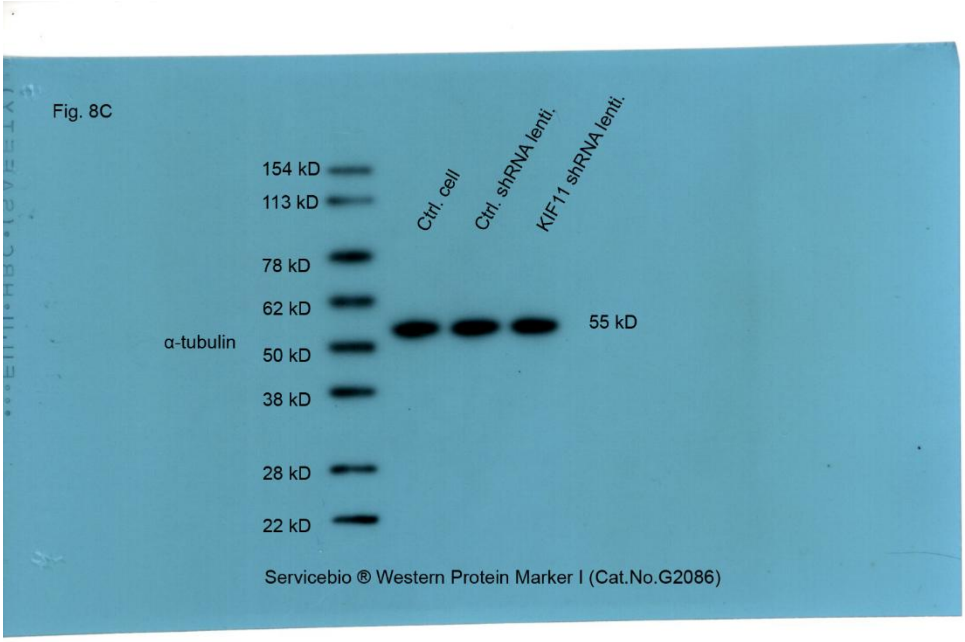
Fig. 8C
154 kD
113 kD
78 kD
62 kD
50 kD
38 kD
28 kD
22 kD
Ctrl. cell
Ctrl. shRNA lenti.
KIF11 shRNA lenti.
α-tubulin
55 kD
Servicebio ® Western Protein Marker I (Cat.No.G2086)

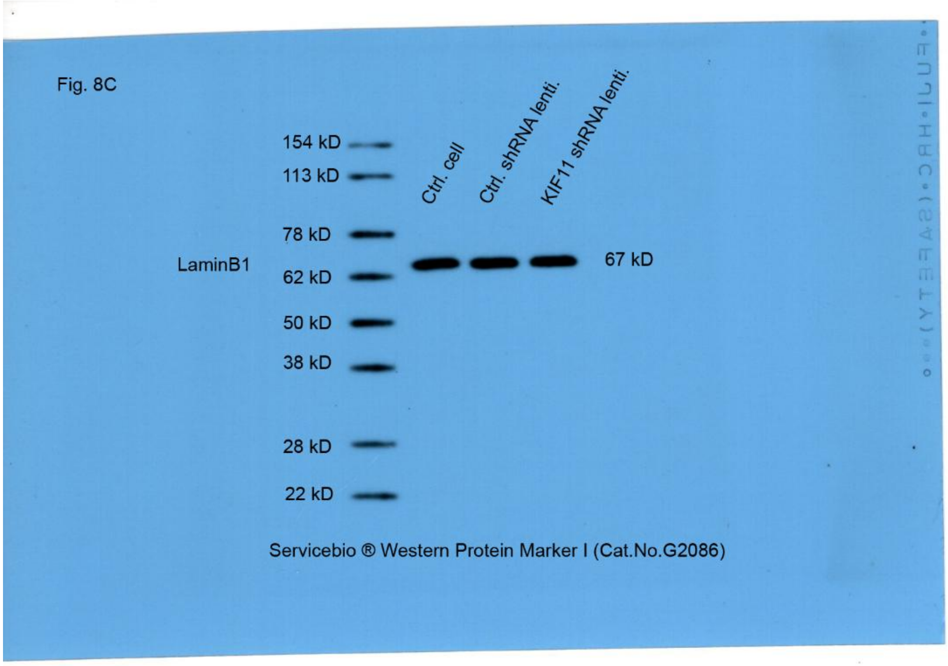
Fig. 8C
LaminB1
154 kD
113 kD
78 kD
62 kD
50 kD
38 kD
28 kD
22 kD
Ctrl. cell
Ctrl. shRNA lenti.
KIF11 shRNA lenti.
67 kD
Servicebio ® Western Protein Marker I (Cat.No.G2086)

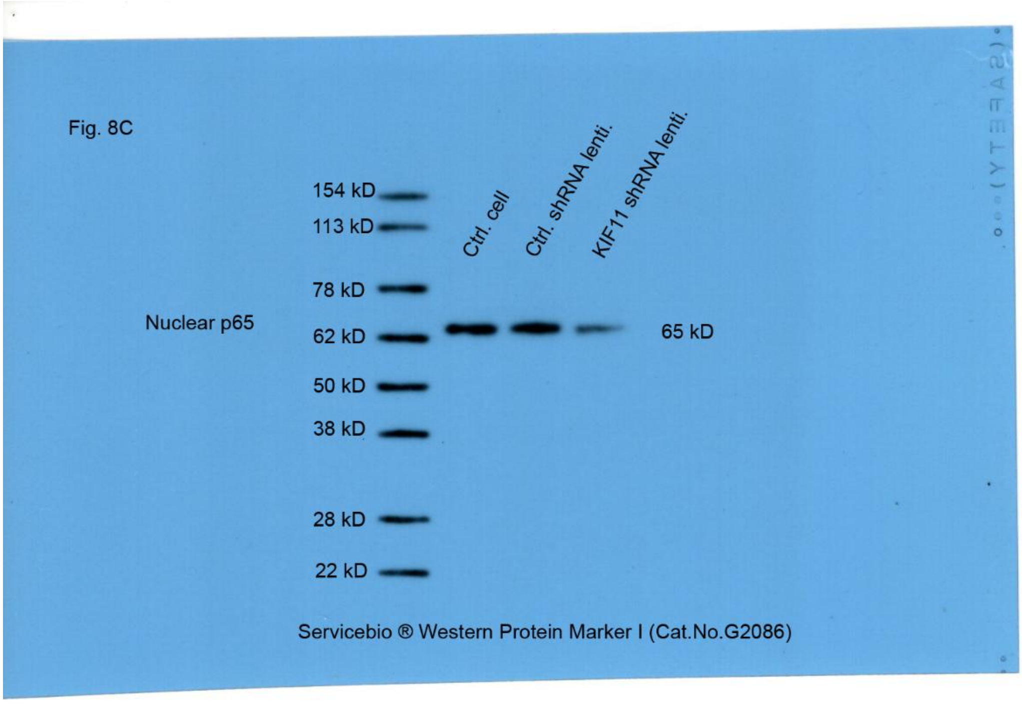
Fig. 8C
Nuclear p65
154 kD
113 kD
78 kD
62 kD
50 kD
38 kD
28 kD
22 kD
Ctrl. cell
Ctrl. shRNA lenti.
KIF11 shRNA lenti.
65 kD
Servicebio ® Western Protein Marker I (Cat.No.G2086)

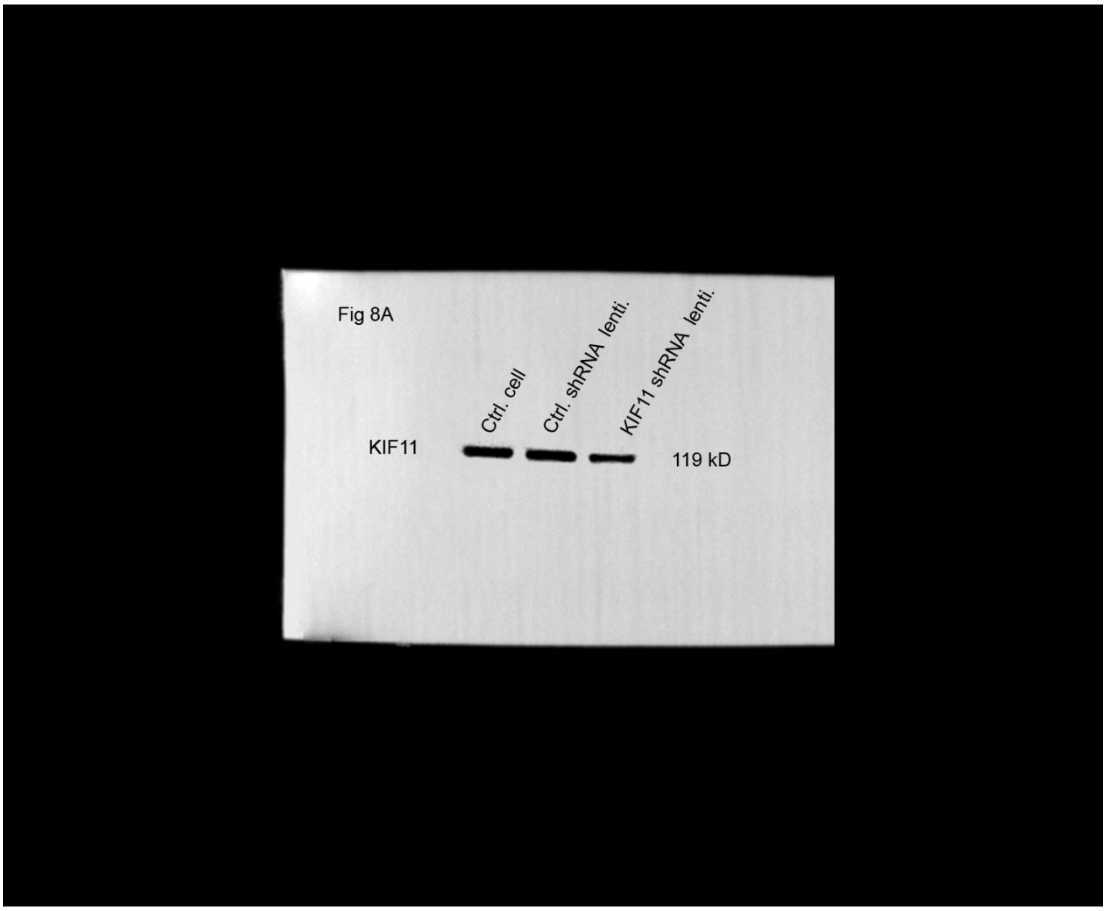
Fig 8A
Ctrl. cell
Ctrl. shRNA lenti.
KIF11 shRNA lenti.
KIF11
119 kD

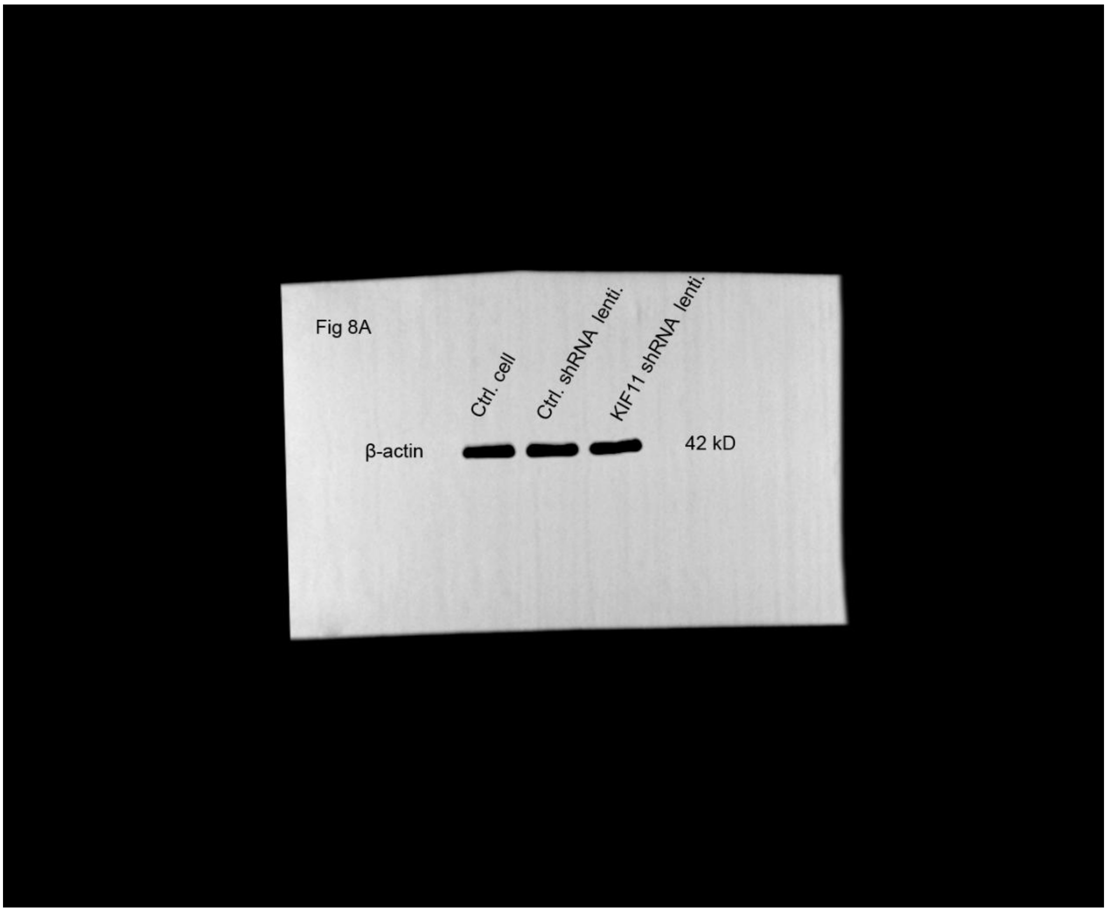
Fig 8A
Ctrl. cell
Ctrl. shRNA lenti.
KIF11 shRNA lenti.
β-actin
42 kD
